# In the Mists of a Fungal Metabolite: An Unexpected Reaction of 2,4,5-Trimethoxyphenylglyoxylic Acid

**DOI:** 10.3390/molecules25081978

**Published:** 2020-04-23

**Authors:** Immo Serbian, Anne Loesche, Sven Sommerwerk, Phil Liebing, Dieter Ströhl, René Csuk

**Affiliations:** 1 Martin-Luther-University Halle-Wittenberg, Organic Chemistry, Kurt-Mothes-Str. 2, D-06120 Halle (Saale), Germany; immo.serbian@chemie.uni-halle.de (I.S.); Anne.Loesche@web.de (A.L.); Svensommerwerk@gmx.de (S.S.); dieter.stroehl@chemie.uni-halle.de (D.S.); 2 Otto von Guericke Universität Magdeburg, Chemisches Institut, Universitätsplatz 2, D-39106 Magdeburg, Germany; Phil.Liebing@ovgu.de

**Keywords:** phenylglyoxylic acid, retro-Friedel–Crafts, fungi, *p*-quinone methide

## Abstract

The reactions of phenylglyoxylic acids during the synthesis and biological evaluation of fungal metabolites led to the discovery of hitherto unknown compounds with a *p*-quinone methide (*p*-QM) structure. The formation of these *p*-QMs using ^13^C-labelled starting materials revealed a key-step of this reaction being a retro-Friedel–Crafts alkylation.

## 1. Introduction

**Figure 1:**
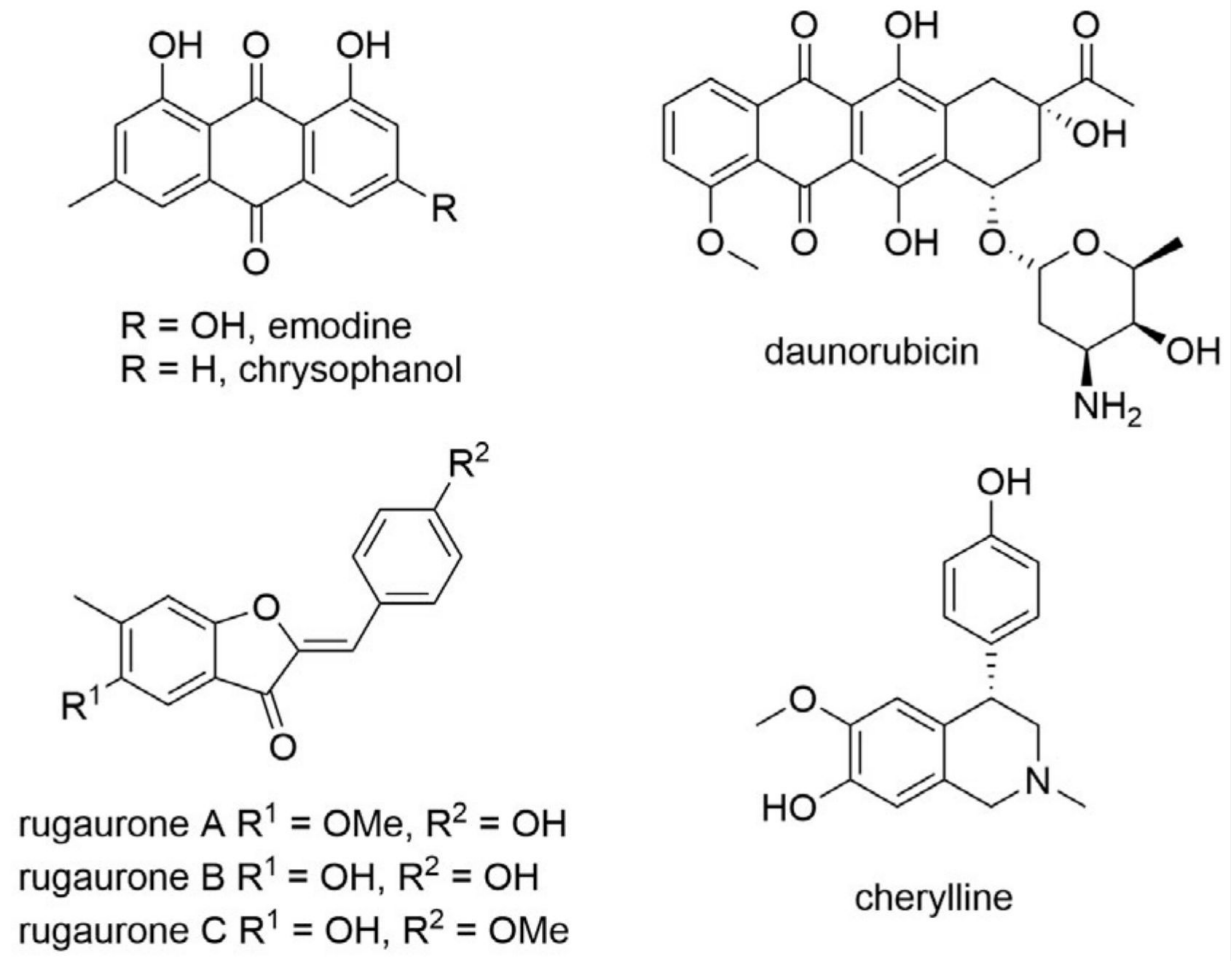
Structure emodine, chrysophanol and daunorubicin isolated from fungi or lichens and *p*-quinone methide (*p*-QM) derived natural products rugaurones A–C and cherylline.

While the secondary metabolites of plants have been studied very intensively, the metabolites formed in fungi and especially of lichens came only recently in the focus of increased scientific interest [1,2,3]. Furthermore, structures similar to 1,3,8-trihydroxy-6-methyl-anthracene-9,10-dione (Figure 1, emodine) or 1,8-dihydroxy-3-methyl-anthracene-9,10-dione (chrysophanol) from fungi have also been isolated from lichens [4,5,6]. Many of these compounds are cytotoxic. For example, the latter compound blocks the proliferation of colon cancer cells by inhibiting the EGFR/mTor pathway [7,8,9]. Some compounds are similar to “-rubicin” anticancer agents, such as daunorubicin or doxorubicin [4,5,6].

*p*-Quinone methides (*p*-QMs) are highly reactive compounds possessing a broad range of different biologic activities [10]. They have also been discussed as the major decomposition products of catechol estrogen-*o*-quinones [11,12,13]. They are well known intermediates in the biosynthesis of natural products. Their high reactivity and potential has also been exploited in the total synthesis of some natural products such as the flavonoids rugaurone A–C [14] cherylline [15] and 20-deoxy-elansolid B1 [16]. Furthermore, the reactions of *p*-QMs have extensively been studied to generate compounds of pharmaceutical interest [17,18,19,20] and quite recently they were used as starting materials for an organo-catalytic asymmetric α-alkylation of aldehydes [21].

During our research on metabolites from fungi and lichens we came across the chemical properties and reactions of phenylglyoxylic acids [22]. Thereby, we encountered several unexpected and unprecedented reactions yielding 3,3-diaryl substituted benzofuranones which undergo retro-Friedel–Crafts alkylation in hydrochloric acid forming the *p*-QM structure. These molecules are similar to 3,3-diaryloxoindoles and 3,3-substituted oxoindoles that have lately been studied quite extensively yielding pharmacological interesting molecules [23,24,25,26,27,28]; they are also known intermediates from isatines and a prominent motif in natural product products, as for example in azonazine [29]. In contrast to isatines, benzofuran-2,3-diones are widely overlooked [28,30]. This was another reason to provide an access to this rare structural motif.

## 2. Results and Discussion

**Scheme 1:**
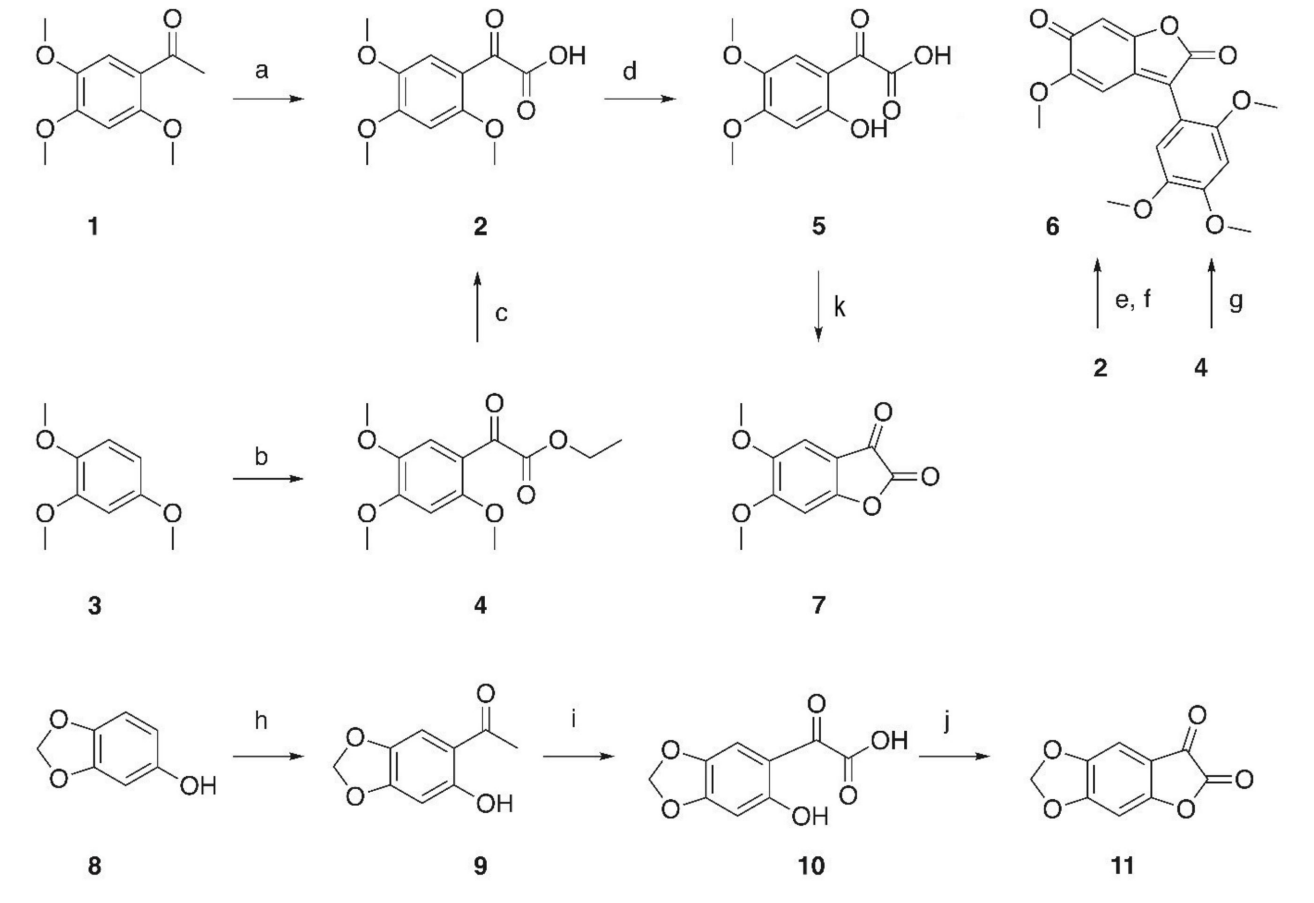
Reactions and conditions: (**a**) SeO_2_, pyridine, 80 °C, 4 h, 57%; (**b**) TiCl_4_, ClC(=O)CO_2_Et, DCM, −20 °C, 2 h, 94%; (**c**) KOH, MeOH, H_2_O, r.t. 99%; (**d**) AlCl_3_, DCM, microwave, 50 °C, 3 h, 13%; (**e**) aq. HCl, 40 °C, 3 h, 56%; (**f**) **3**, HCl, 40 °C, 4 h, 96%; (**g**) aq. HCl, 40 °C, 4 h, 49%; (**h**) Ac_2_O, BF_3_^.^Et_2_O, 85 °C, 1 h, 66%; (**i**) SeO_2_, pyridine, 80 °C, 20 h, 44%; (**j**) DCM, oxalyl chloride, DMF, r.t. 14 h, 36%; (**k**) DCM, oxalyl chloride, DMF, 0 °C, 2h, traces.

Oxidation of 2,4,5-trimethoxy-acetophenone (**1**, Scheme 1) with SeO_2_ [31] gave 2,4,5-trimethoxyphenylglyoxylic acid (**2**); this compound has previously been isolated from the fungus *Polyporus tumulosus* Cooke [22,32]. As an alternative, a Friedel–Crafts acylation of 2,4,5-trimethoxybenzene (**3**) in the presence of TiCl_4_ gave a 94% yield of ester **4** [33,34,35] whose hydrolysis with methanolic KOH for 2 h resulted in an almost quantitative yield of **2**. Partial deprotection of the methoxy-groups with AlCl_3_ of **2** yielded **5** albeit in low yields, while demethylation with either hydrobromic or hydrochloric acid yielded **6**, a red colored solid in almost quantitative yield. A major issue in these reactions is the instability of the α-keto-acids that are readily decarboxylated [22,36]. These findings parallel previous reports for trimethoxy-substituted aromatic compounds [36]. Furthermore, α-keto acids were used for the synthesis of oxadiazolopyrazines—selective antibacterial agents against *Haemophilus influenzae* [37]. Attempts to cyclize **5** under a variety of different conditions invariably led to the formation of **6** in moderate to excellent yields. The formation of **7** was only observed to a rather minor extend by ESI–MS.

Friedel–Crafts acylation of sesamol (**8**) with acetic anhydride/BF_3_ gave **9** [38,39,40,41] in 66% isolated yield whose oxidation with SeO_2_ in pyridine yielded **10**. Treatment of **10** with oxalyl chloride in DCM in the presence of DMF gave a moderate yield of **11** but—interestingly enough - no red-colored by-products (being analogous to **6**) were observed during these reactions.

**Scheme 2:**
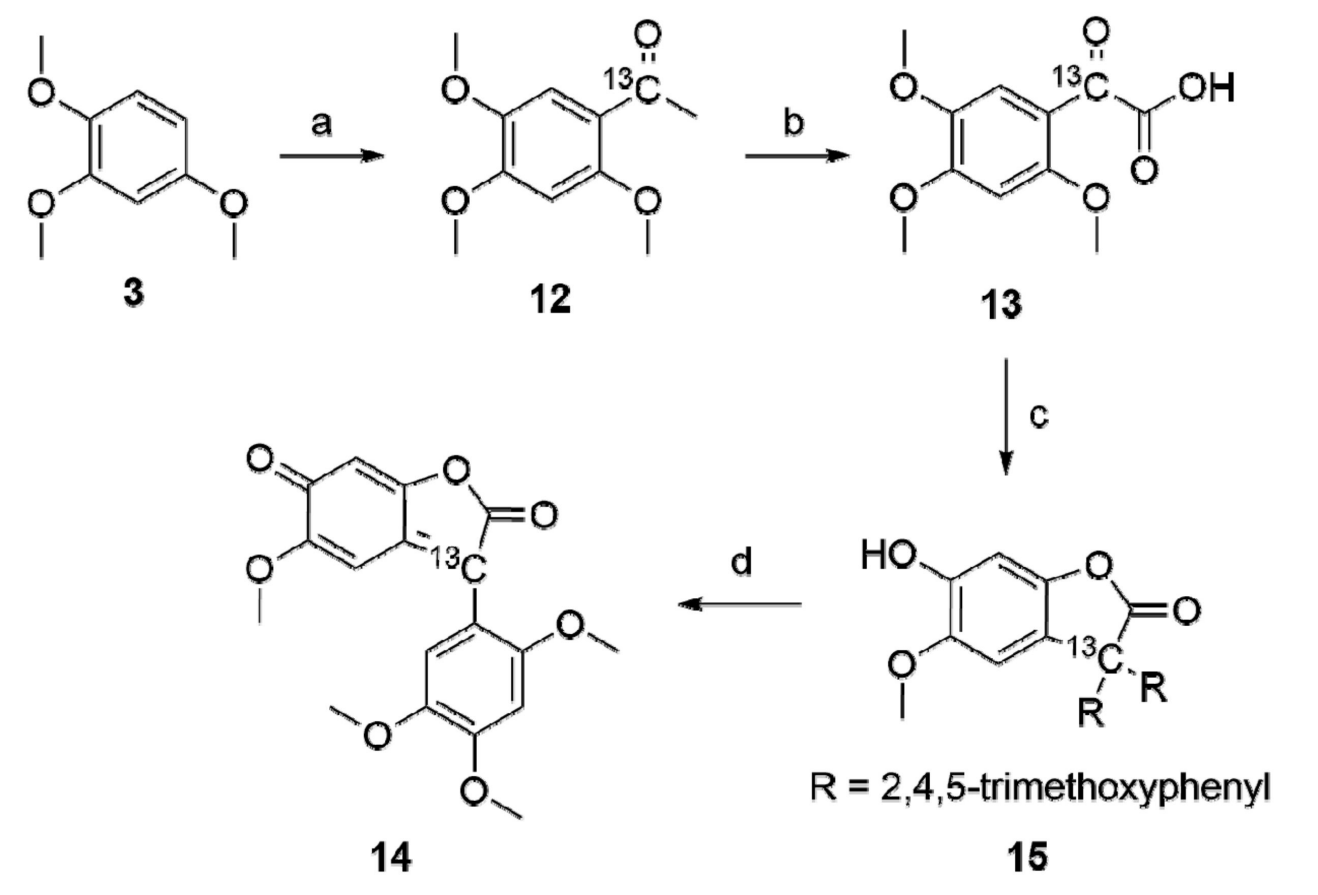
Reactions and conditions: (**a**) AcCl, TiCl_4_, DCM, −20 °C, 2 h, 95%; b) SeO_2_, pyridine, 80 °C, 4 h, 53%; (**c**) aq. HCl, 0 °C, 2 h, 3%; (**d**) aq. HCl, Δ,2 h, 48%.

To gain a deeper insight in the structure and formation of **6**, ^13^C-labeling experiments were called for. Thus, trimethoxybenzene (**3**) was allowed to react with ^13^C-labeled acetylchloride (Scheme 2) in the presence of TiCl_4_ and a 97% yield of ^13^C-labeled **1** (**12**) was obtained. From its SeO_2_ oxidation compound ^13^C-labeled **2** (**13**) was obtained in 53% yield.

To elucidate the structure of **6**, a combination of different analytical techniques had to be applied. An ESI/MS of **6** in MeOH showed a *m/z* = 345 [M + H]^+^ corresponding to a molecular composition of C_18_H_16_O_7_ and indicating a “condensation reaction” of **2** having taken place. The ^1^H-NMR spectra showed the presence of four methoxy groups between δ = 3.81 and 3.97 ppm and four aromatic hydrogens between δ = 6.06 and 7.26 ppm, respectively. The material obtained from the ^13^C-labeled starting material showed only one labeled carbon in the product as indicated by ESI–MS *m/z* = 346 for [M + H]^+^.

**Figure 2:**
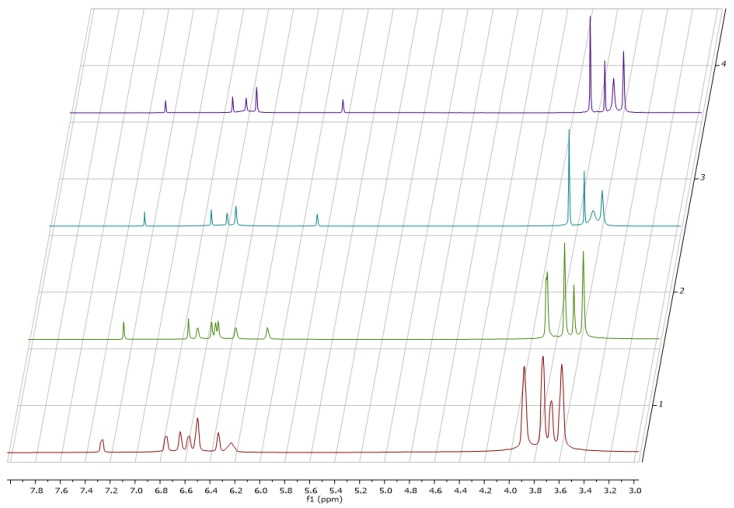
^1^H-NMR Spectra of the intermediate **15**; temperatures: **violet**, 40 °C; **cyan**, 27 °C; **green**, −30 °C; red, −50 °C.

**Figure 3:**
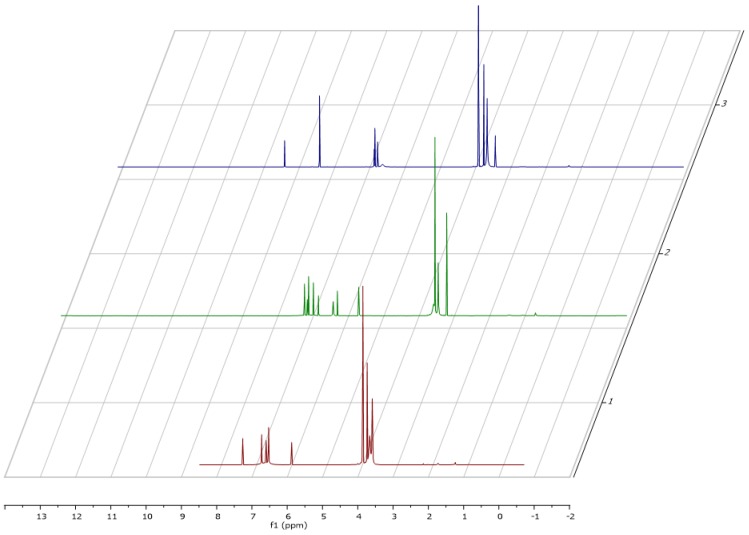
NMR Spectra of the intermediate **15** in different solvents; CDCl_3_ (**red**), toluene-d_8_ (**green**), DMSO-d_6_ (**blue**) at room temperature.

Reaction of **2** with AlCl_3_ or **13** with hydrochloric acid at low temperatures, however, gave access to an intermediate **15** (from **13**); upon warming this reaction mixture, **15** could not be detected any longer but **6** (from **2**) or **14** (from **13**) was formed. For compound **15**, different temperatures (−50, −30, 27 and 40 °C) were applied (Figure 2) and the ^1^H-NMR spectroscopy revealed a change in the spectra. At room temperature (Figure 2, cyan) an extensive line broadening of one of the methoxy groups (δ = 3.62 ppm) was observed. In addition, extensive line broadening was seen in the aromatic region. Integration of the signals suggested the presence of seven methoxy groups. Temperature dependent NMR spectroscopy revealed that the line broadening observed at room temperature is due to the presence of a rotational barrier. Furthermore, the NMR spectra of **15** strongly depend on the used solvent (Figure 3). For example, the aromatic protons in **15** are severely shifted to lower field upon using deuterated toluene as the solvent probably due to an aromatic solvent induced shift behavior. An ESI–MS of **15** showed a quasimolecular ion [M + H]^+^ *m/z* = 513 corresponding well to the proposed structure. NMR spectra of the products are depicted in the Supplementary File.

**Figure 4:**
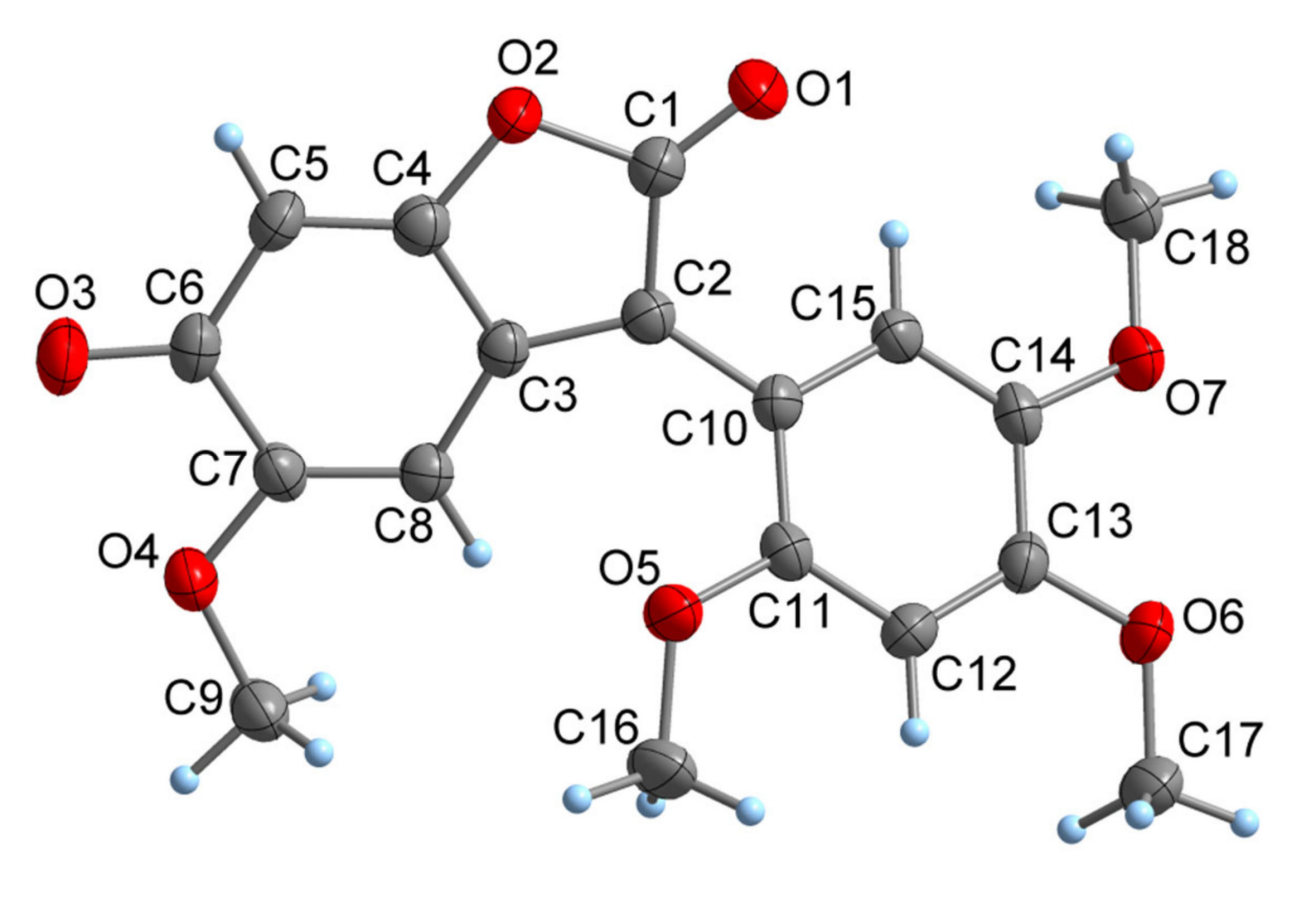
The molecular structure of compound **6** in the crystal. Displacement ellipsoids of C and O atoms drawn at the 50% probability level, H atoms as spheres of arbitrary size.

As **6** crystallizes readily from ethyl acetate, crystals suitable for a single crystal X ray analysis could be obtained. The crystal structure of the black orthorhombic prisms (space group P2_1_2_1_2_1_) is consistent with the NMR data comprising C_18_H_16_O_7_ molecules. The molecular structure is depicted in Figure 4.

**Figure 5:**
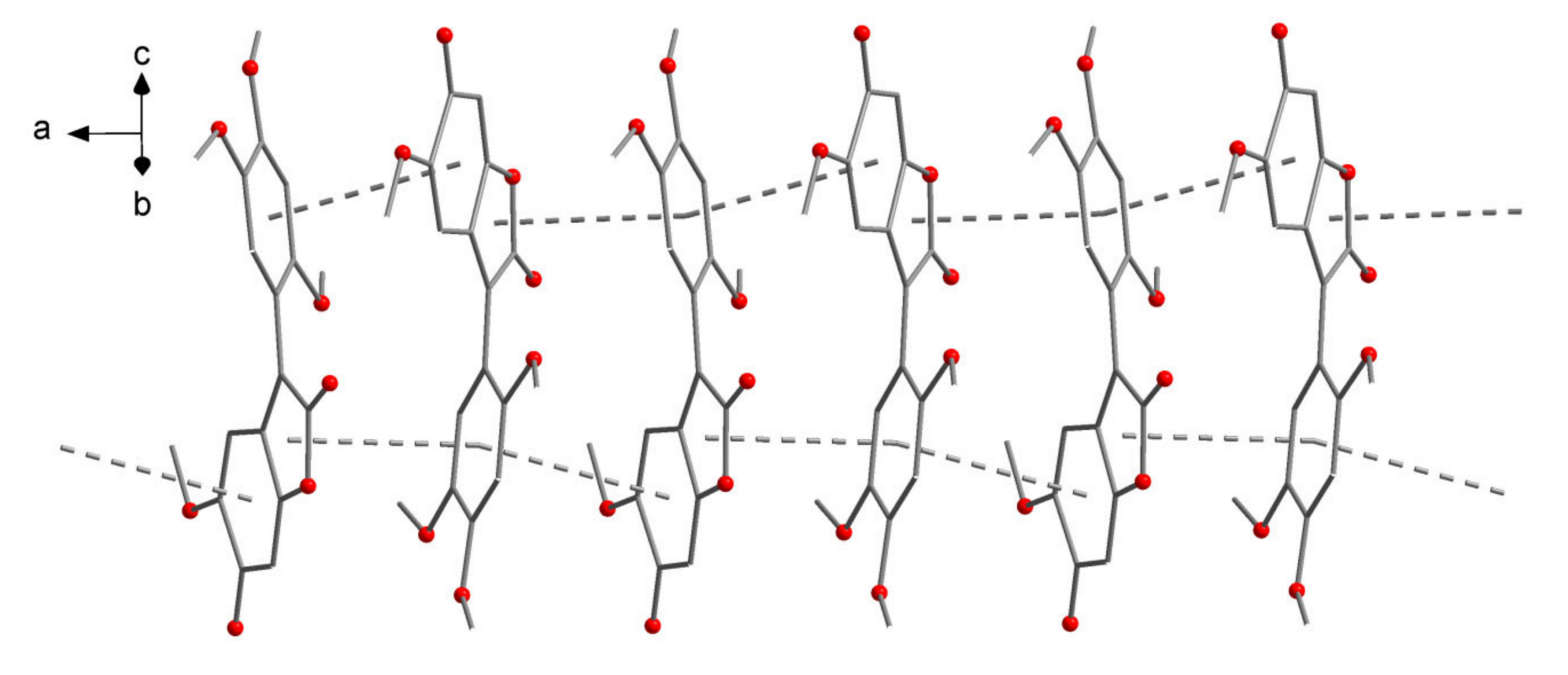
Intermolecular π interactions in compound **6**, resulting in a one-dimensional supramolecular structure extending along the crystallographic *a* axis.

The bond lengths within the benzofurane-derived bicyclic system (C1–C8, O2) cover a wide range of 133.2(3)–150.1(4) pm. C1–C2 (147.4(4) pm), C3–C4 (144.0(4) pm), C3–C8 (142.8(4) pm), C5–C6 (145.8(4) pm), C6-C7 (150.1(4) pm), C1–O2 (140.6(3) pm) and C4–O2 (138.2(3) pm) can be described as single bonds, while C2–C3 (136.0(3) pm), C4–C5 (133.2(3) pm) and C7–C8 (134.2(3) pm) have double bond character. The interconnection between the two ring systems, C2–C10, is a single bond at 147.0(4) pm. The C–C separations within the benzene ring (C10–C15) are in a typical narrow range of 137.3(4)–141.1(3) pm. The molecule is not strictly planar as the two ring systems are twisted around the C2–C10 vector about 41.0(1)°. In the crystal, the molecules are stacked together to a one-dimensional supramolecular array by π interactions (Figure 5), with the closest intermolecular contacts being C13∙∙∙O2 (342.2(4) pm) and C8∙∙∙C15 (344.7(4) pm). The distances between the ring centroids are 356.81(3) pm ((C1–C4, O2)∙∙∙(C10–C15)) and 369.58(3) pm ((C3–C8)∙∙∙(C10–C15)), respectively.

**Scheme 3:**
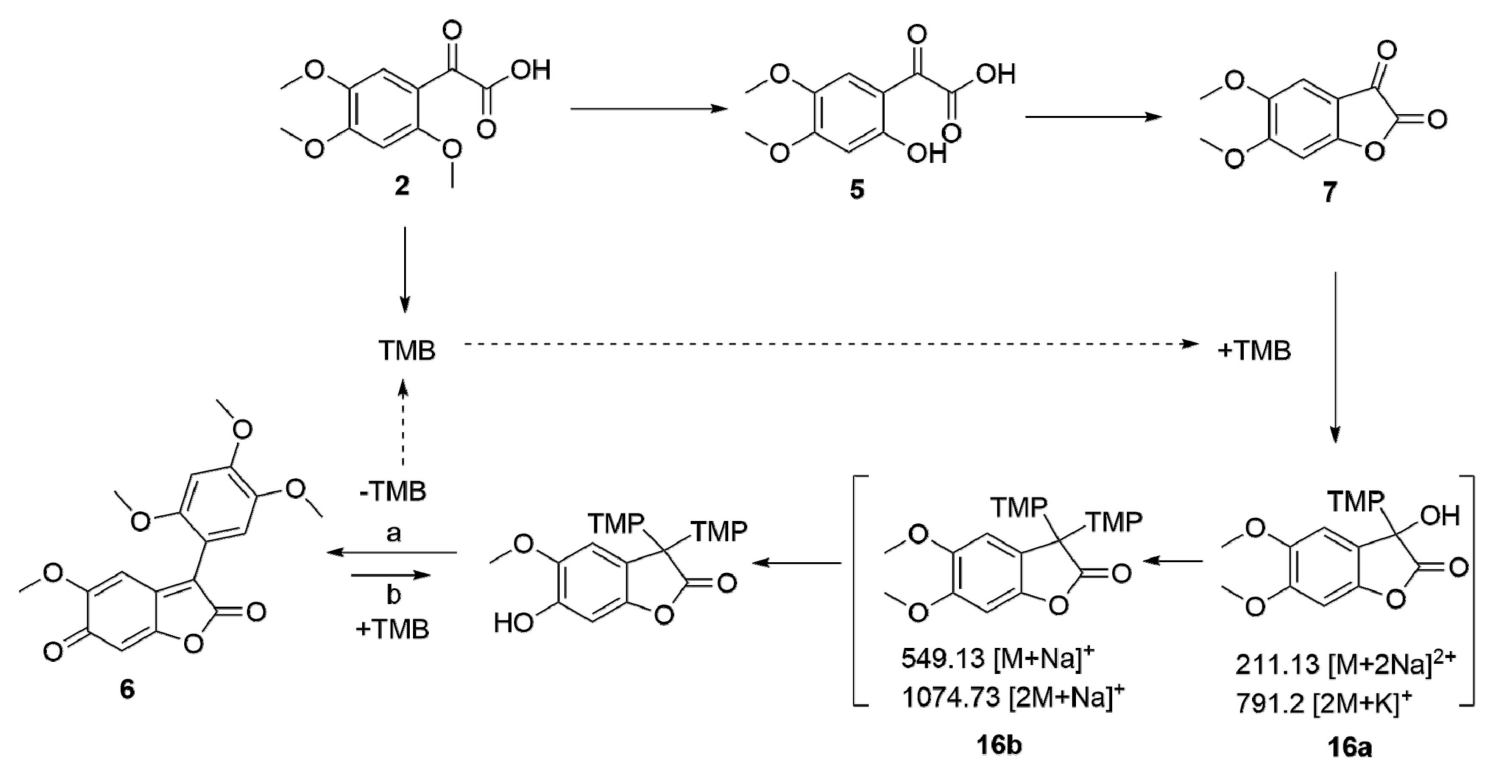
Proposed mechanism for the formation of **6** (TMP = 2,4,5-trimethoxyphenyl; TMB = 1,2,4-trimethoxybenzene).

While the reaction of **2** with trifluoroacetic acid (TFA) in dry DCM did not lead to the formation of **6** and with conc. sulfuric or phosphoric acid only low yields (<10%) were observed, the reaction of **2** with HBr (48%) gave 42% of **6** and with conc. HCl (37%) an 56% yield was observed. Reaction of **2** with 37% HCl in the presence of 5 equivalents 1,2,4-trimethoxybenzene finally led to a 96% yield of **6**. From these observations as well as from the ^13^C-labeling experiments (vide supra), a tentative mechanism for this reaction was deduced (as depicted in Scheme 3). In the course of the reaction of **2** with TFA in dry DCM an intermediate **16a** was observed. ESI-MS experiments showed the presence of a quasimolecular ions *m/z* = 211.1 ([M + 2Na]^2+^) and *m/z* = 791.2 ([2M + K]^+^) corresponding to M = 376. Subsequently from **16a** intermediate **16b** is formed; the latter compound was detected in ESI–MS spectra showing *m/z* = 549.13 ([M + Na]^+^) and 1074.73 ([2M + Na]^+^). Friedel–Crafts reactions are known to be reversible [42,43,44,45,46,47,48] and compound **6** is very insoluble in the reaction mixture thus explaining the very high yield of **6** in these reactions. Previously, a retro Friedel–Crafts alkylation was used to access a fungal pigment from *Peniophora sanguinea* Bres [13].

To verify these assumptions, to a solution of **6** in dry 2M HCl (in ethyl acetate) **3** was added in 10-fold excess and within several hours of stirring at room temperature, the reaction mixture turned from black to slightly red. ESI–MS investigations of this reaction mixture showed the formation of **15** (*m/z* = 513, [M + H]^+^).

**Scheme 4:**
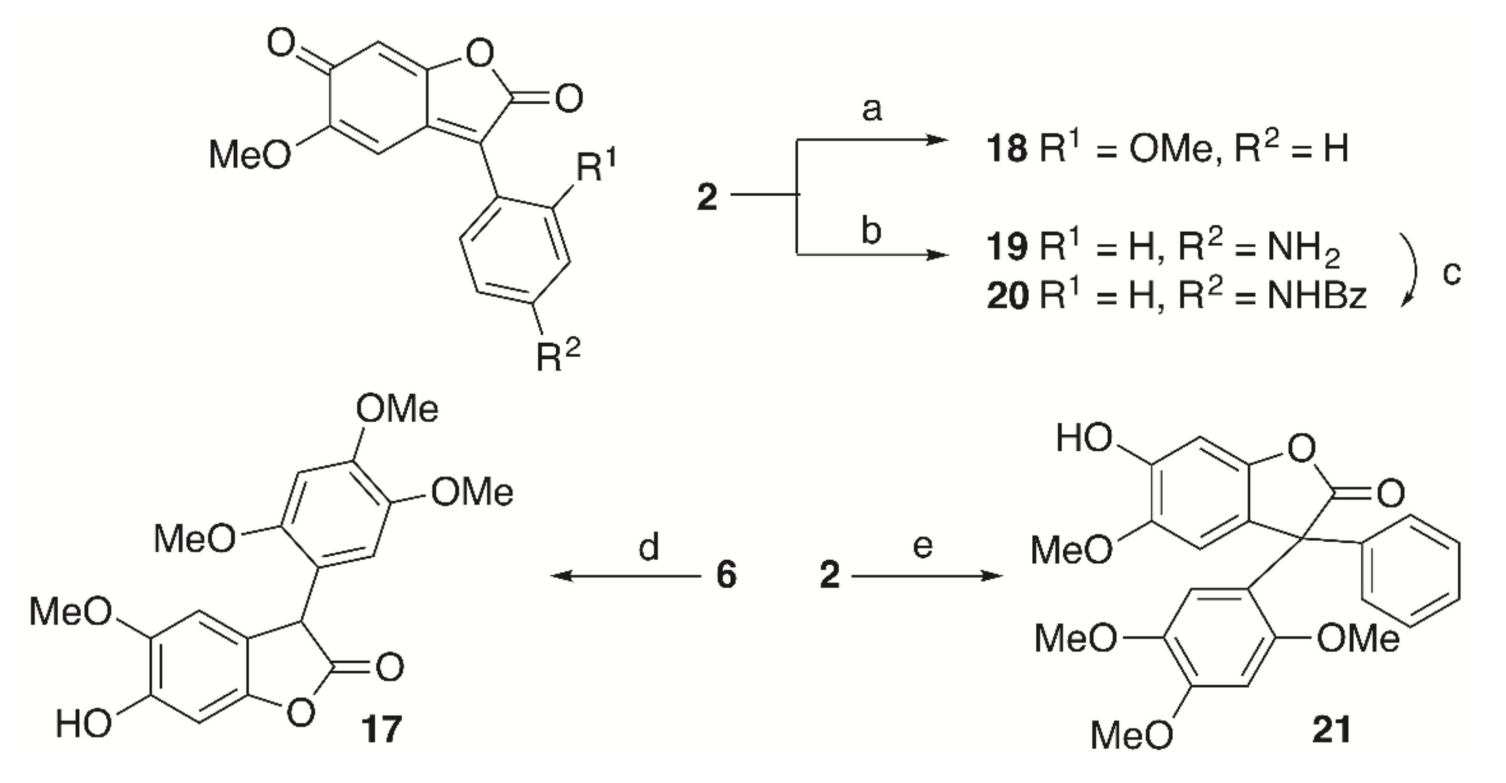
Reactions and conditions: (**a**) anisole, HCl, 40 °C, 4 h, 74%; (**b**) Boc-aniline, HCl, 40 °C, 4 h, 43%; (**c**) BzCl, NEt_3_, DCM, r.t. 6 h, 69%; (**d**) Zn/HCl, 0 °C, 4 h, 43%; (**e**) benzene, HCl, 2 h, 18%.

Treatment of **6** with Zn/HCl led to the formation of **17**. To have a first insight into the scope of this reaction (Scheme 4), **2** was allowed to react with anisole or BOC-aniline in the presence of hydrochloric acid and products **18** and **19** were obtained, respectively. Benzoylation of **19** gave **20**. Interestingly enough, reaction of **2** with benzene in the presence of hydrochloric acid furnished a 18% yield of **21**.

## 3. Materials and Methods

NMR spectra were recorded using the Varian spectrometers (Varian GmbH, Darmstadt, Germany) Gemini 2000 or Unity 500 (δ given in ppm, *J* in Hz; typical experiments: APT, H–H-COSY, HMBC, HSQC, NOESY), MS spectra were taken on a Finnigan MAT LCQ 7000 (electrospray, voltage 4.1 kV, sheath gas nitrogen) instrument. TLC was performed on silica gel (Merck 5554, detection with cerium molybdate reagent); melting points are uncorrected (Leica hot stage microscope, Leica GmbH, Wetzlar, Germany) and elemental analyses were performed on a Foss-Heraeus Vario EL (CHNS, Elementar analysensysteme GmbH, Langenselbold, Germany) unit. IR spectra were recorded on a Perkin Elmer (Perkin Elmer Deutschland, Berlin, Germany) FT-IR spectrometer Spectrum 1000 or on a Perkin-Elmer Spectrum Two (UATR Two Unit). The solvents were dried according to usual procedures. Crystallographic Data were deposited at the Cambridge Crystallographic Data Center with the depository number CCDC 1569146. The data are available free of charge at www.ccdc.cam.ac.uk/products/csd upon request.

## 4. Experimental

### 4.1. 2,4,5-Trimethoxyphenylglyoxylic Acid (***2***)

*From***1**. A suspension of 2,4,5-trimethoxybenzene (1, 840 mg, 4.00 mmol) and selenium dioxide (800 mg, 7.21 mmol) in pyridine (5 mL) was stirred for 4 h at 80 °C. The mixture was poured into NaOH (100 mL, 0.05 m) and extracted with EtOAc (3 × 50 mL). The aqueous phase was acidified with 2 m hydrochloric acid and extracted with EtOAc (3 × 50 mL). The combined organic extracts were washed with brine, dried (MgSO_4_), the solvent was evaporated and the residue re-crystallized from EtOAc to yield **2** (550 mg, 57%) as a yellow solid.

*From***4**. To a solution of 4 (1.0 g, 3.73 mmol) in methanol (50 mL), powdered KOH (1.5 g, 26.7 mmol) was added and the mixture was stirred for 2 h. Water (100 mL) was added and the aqueous phase was extracted with chloroform (3 × 70 mL). The combined organic phases were dried (MgSO_4_) and the solvent was removed to afford **2** (886 mg, 99%) as a yellow solid; an analytical sample showed m.p. 182–185 °C (lit.:[49] 186 °C); R_F_ = 0.35 (SiO_2_, CHCl_3_/MeOH; 2:1); IR (KBr): ν = 3432br, 1736s, 1610s, 1517s, 1480m, 1423w, 1384w, 1288s, 1288s, 1230s, 1190m, 1142s, 1018s cm^−1^; UV-Vis (MeOH): λ_max_ (log ε) = 237 (4.22), 279 (4.09), 341 (4.01) nm; ^1^H-NMR (400 MHz, DMSO-*d_6_*): δ = 7.22 (s, 1H, 8-H), 6.82 (s, 1H, 5-H), 3.92 (s, 3H, 9-H), 3.84 (s, 3H, 10-H), 3.76 (s, 3H, 11-H) ppm; ^13^C-NMR (100 MHz, DMSO-d_6_): δ = 186.0 (C-2), 167.6 (C-1), 157.2 (C-6), 156.4 (C-4), 143.7 (C-7), 113.1 (C-3), 110.2 (C-8), 98.1 (C-5), 57.1 (C-10), 56.2 (C-9), 55.8 (C-11) ppm; MS (ESI, MeOH): *m/z* (%) = 238.9 ([M − H]^−^, 100), 478.8 ([2M − H]^−^, 8), 501.0 ([2M − 2H + Na]^−^, 3); analysis calcd for C_11_H_12_O_6_ (240.21): C 55.00, H 5.04; found: C 54.79, H 5.18.

### 4.2. 2,4,5-Trimethoxyphenyl-glyoxylic Acid Ethyl-ester (***4***)

To a solution of 1,2,4-trimethoxybenzene (3, 5.0 g, 29.7 mmol) in DCM (200 mL) at −20 °C, TiCl_4_ (3.6 mL, 32.8 mmol) was added followed by slowly adding ethyl chlorooxoacetate (3.5 mL, 31.3 mmol) and the mixture was stirred for 2 h. Aqueous work-up (2M HCl) followed by extraction with chloroform (3 × 100 mL) and column chromatography (SiO_2_, hexanes/CHCl_3_, 1:1) gave **4** (7.48 g, 94%) as pale yellow crystalline solid; m.p. 89–91 °C (lit.:[50] 89.5 °C); R*_F_* = 0.78 (SiO_2_, hexanes/CHCl_3_); IR (ATR): ν = 3004w, 2991w, 2943w, 2908w, 2840w, 1732m, 1633m, 1599s, 1581m, 1513s, 1482m, 1461m, 1450m, 1440m, 1418m, 1390w, 1371m, 1298s, 1268s, 1259s, 1228s, 1218s, 1191s, 1179s, 1141vs, 1112m, 1038m, 1021s, 1007s, 954m, 862m, 816m, 805m, 770m, 765m, 743m, 710m, 655m cm^−1^; UV-Vis (methanol): λ_max_ (logε) 237 (3.52), 281 (3.40), 346 (3.34) nm;^1^H-NMR (400 MHz, CDCl_3_) δ = 7.38 (s, 1H, 2′-H), 6.46 (s, 1H, 5′-H), 4.36 (q, *J* = 7.1 Hz, 2H, Et_CH2_), 3.95 (s, 3H, OMe(6′)), 3.87 (s, 3H, OMe(3′)), 3.84 (s, 3H, OMe(4′)), 1.38 (t, *J* = 7.1 Hz, 3H, Et_CH3_) ppm; ^13^C-NMR (101 MHz, CDCl_3_) δ = 184.8 (C-2), 166.1 (C-1), 157.3 (C-4‘), 156.5 (C-6‘), 144.2 (C-3‘), 114.2 (C-1‘), 111.0 (C-2‘), 96.5 (C-5‘), 61.6 (C-Et_CH2_), 56.9 (OMe(C4‘)), 56.3 (OMe(C3‘)), 56.3 (OMe(C2‘)), 14.1 (CEt_CH3_) ppm; MS (ESI, MeOH): *m/z* (%) = 269.1 ([M + H]^+^, 100); analysis calcd for C_13_H_16_O_6_ (268.26): C 58.20, H 6.01; found: C 57.95, H 6.22.

### 4.3. 2-Hydroxy-4,5-dimethoxyphenyl-glyoxylic Acid (***5***)

A solution of AlCl_3_ (166 mg, 1.25 mmol) and 2 (150 mg, 0.62 mmol) in DCM (5 mL) was heated for 3 h under microwave irradiation at 50 °C. Usual aqueous work-up (aq. HCl, 80 mL, 0.125 m) followed by extraction (ethyl acetate, 2 × 50 mL) and re-crystallization from chloroform gave 5 (17 mg, 13%) as yellow solid; m.p. 163–165 °C (lit:[22] 142–144 °C); R*_F_* = 0.25 (SiO_2_, CHCl_3_/MeOH 2:1); IR (KBr): ν = 3150br, 1713s, 1573s, 1526s, 1482m, 1400m, 1352w, 1308m, 1267s, 1240m, 1205s, 1181m, 1165s, 1035m cm^−1^; UV-Vis (MeOH): λ_max_ (log ε = 238 (4.07), 283 (3.99), 344 (3.93) nm; ^1^H-NMR (400 MHz, DMSO-d_6_): δ = 7.24 (s, 1H, 8-H), 6.61 (s, 1H, 5-H), 3.78 (s, 3H, 10-H), 3.74 (s, 3H, 9-H) ppm; ^13^C-NMR (101 MHz, DMSO-d_6_): δ = 185.6 (C-2), 167.8 (C-1), 157.1 (C-6), 155.4 (C-4), 142.8 (C-7), 112.3 (C-3), 111.2 (C-8), 100.7 (C-5), 56.6 (C-10), 56.0 (C-9) ppm; MS (ESI, MeOH): *m/z* (%) = 224.9 ([M − H]^−^, 100), 472.9 ([2M − 2H + Na]^−^, 16); analysis calcd for C_10_H_10_O_6_ (226.18): C 53.10, H 4.46; found: C 52.96, H 4.60.

### 4.4. 5-Methoxy-3-(2,4,5-trimethoxyphenyl)benzofuran-2,6-dione (***6***)

*Method A.* Compound **2** (250 mg, 1.04 mmol) was suspended in conc. hydrochloric acid (25 mL) and the mixture was heated to 40 °C for 3 h, diluted with water (25 mL) and extracted with chloroform (3 × 25 mL). The organic extracts were dried (MgSO_4_), the solvent was removed and the remaining residue was subjected to column chromatography (SiO_2_, CHCl_3_/MeOH 9:1) to afford **6** (199 mg, 56%) as a dark red solid.

*Method B.* Compound **2** (200 mg, 0.83 mmol) and 1,2,4-trimethoxybenzene (0.62 mL, 4.16 mmol) were suspended in conc. hydrochloric acid (25 mL) and the mixture was heated to 40 °C for 4 h, diluted with water (25 mL) and extracted with chloroform (3 × 25 mL). The organic extracts were dried (MgSO_4_), the solvent was removed and the remaining residue was subjected to column chromatography (SiO_2_, CHCl_3_/MeOH 9:1) to afford **6** (274 mg, 96%) as a dark red solid.

*Method C.* Compound **4** (1.0 g, 3.73 mmol) was suspended in hydrochloric acid (50 mL) and heated to 40 °C for 4 h; workup as described above gave **6** (636 mg, 49%) as dark red solid; m.p. 238–239 °C; R*_F_* = 0.68 (SiO_2_, CHCl_3_/MeOH 9:1); IR (ATR): ν = 2958w, 2941w, 2841w, 1772s, 1735w, 1643s, 1610m, 1588vs, 1559s, 1527m, 1517m, 1506m, 1461s, 1454s, 1437m, 1416m, 1388m, 1350s, 1316w, 1274s, 1234vs, 1223s, 1209vs, 1192s, 1180vs, 1167vs, 1134s, 1052w, 1040m, 1023s, 1001s, 985s, 934s, 864s, 854s, 844s, 822m, 814m, 778m, 774m, 766m 701m, 696m, 669m cm^−1^, UV–Vis (MeOH): λ_max_ (log ε = 311 (3.31), 357 (3.08), 498 (3.00) nm; ^1^H-NMR (400 MHz, CDCl_3_): δ = 7.09 (s, 1H, 6′-H), 6.61 (s, 1H, 3′-H), 6.36 (s, 1H, 4-H), 6.06 (s, 1H, 7-H), 3.97 (s, 3H, OMe(4′)), 3.88 (s, 3H, OMe(5′), 3.87 (s, 3H, OMe(2′)), 3.82 (s, 3H, OMe(5)) ppm; ^13^C NMR (101 MHz, CDCl_3_): δ = 181.3 (C-6), 167.4 (C-2), 160.5 (C-7a), 153.82 (C-2′), 152.78 (C-4′), 152.74 (C-5), 143.74 (C-5′), 138.80 (C-3a), 125.59 (C-3), 114.3 (C-6′), 109.3 (C-1′), 104.2 (C-7), 100.7 (C-4), 97.6 (C-3′), 56.8 + 56.6 (OMe(2′) + OMe (5′)), 56.3 (OMe(4′)), 56.3 (OMe(5)) ppm; MS (ESI, MeOH): *m/z* (%) = 345.2 ([M + H]^+^, 100); analysis calcd for C_18_H_16_O_7_ (344.32): C 62.79, H 4.68; found: C 62.55, H 4.83.

### 4.5. 1-(6-Hydroxybenzo[d][1,3]dioxol-5-yl)ethan-1-one (***9***)

To a suspension of sesamol (8, 5.52 g, 40.00 mmol) in acetic anhydride (20 mL, 0.21 mol) at 0 °C BF_3_∙OEt_2_ (10 mL, 81.03 mmol) was added and the reaction mixture was heated at 85 °C for 1 h. An aqueous solution of sodium acetate sol. (satd., 40 mL) was added and the mixture was extracted with diethylether (3 × 50 mL). The combined organic layers were washed with sat. NaHCO_3_ sol. (4 × 100 mL), water (3 × 100 mL), brine (1 × 100 mL) and dried (MgSO_4_). The solvent was removed and recrystallization from ethanol gave **9** (4.74 g, 66%) as an off-white solid; m.p. 111–113 °C (lit.:[51] 114 °C); R*_F_* = 0.81 (SiO_2_, CHCl_3_/MeOH, 9:1); C; IR (KBr): ν = 3446br, 2919m, 1633s, 1485s, 1425s, 1366m, 1322s, 1257s, 1182s, 1118m, 1037s, 958m, 924s, 856m, 844m, 787m, 773m, 540m cm^−1^; UV–Vis (CHCl_3_): λ_max_ (log ε) = 241 (4.16), 278 (3.86), 349 (3.92) nm; ^1^H-NMR (400 MHz, CDCl_3_): δ = 7.04 (s, 1H, 6-H), 6.43 (s, 1H, 3-H), 5.97 (s, 2H, 7-H), 2.51 (s, 3H, 9-H) ppm; ^13^C NMR (100 MHz, CDCl_3_): δ = 202.0 (C-8), 162.2 (C-2), 154.5 (C-4), 140.6 (C-5), 112.4 (C-1), 107.3 (C-6), 102.0 (C-7), 98.8 (C-3), 26.6 (C-9) ppm; MS (ESI, MeOH): *m/z* (%) = 181.1 ([M + H]^+^, 100); analysis calcd for C_9_H_8_O_4_ (344.32): C 62.79, H 4.68; found: C 62.57, H 4.90.

### 4.6. 2-(6-Hydroxybenzo[d][1,3]dioxol-5-yl)-2-oxoacetic Acid (***10***)

Compound **9** (2.5 g, 13.88 mmol) and selenium dioxide (3.08 g, 27.75 mmol) were suspended in dry pyridine (45 mL) and stirred at 80 °C for 20 h. Aqueous work-up (400 mL, 1 m), followed by extraction with EtOAc (8 × 100 mL) and evaporation of the solvent gave **10** as a brownish solid (1.96 g, 67%). An analytical sample was obtained by re-dissolving **10** in EtOAc (100 mL), washing with NaOH (100 mL, 0.2 m), separation of the phases, acidification of the aqueous phase with HCl (2 m, 2.5 mL) followed by extraction with EtOAc (2 × 50 mL). The combined organic extracts were washed with brine, dried (MgSO_4_) and the solvent was removed at room temperature to yield analytically pure **10** (1.27 g, 44%) as an orange solid; m.p. 138–141 °C; R*_F_* = 0.22 (SiO_2_, CHCl_3_/MeOH, 4:1); IR (KBr): ν = 3422br, 3284m, 2925w, 1765m, 1734w, 1616m, 1593m, 1481m, 1416m, 1376w, 1289m, 1237s, 1176m, 1102w, 1038m cm^−1^; UV–vis (MeOH): λ_max_ (log ε) = 246 (4.04), 286 (3.80), 358 (8.87) nm; ^1^H-NMR (400 MHz, DMSO-d_6_): δ = 7.08 (s,1H, 8-H), 6.53 (s, 1H, 5-H), 6.08 (s, 2H, 9-H) ppm; ^13^C-NMR (101 MHz, DMSO-d_6_): δ = 187.9 (C-2), 167,4 (C-1), 159.8 (C-4), 155.3 (C-6), 141.7 (C-7), 111.7 (C-3), 106.6 (C-8), 102.8 (C-9), 98.3 (C-5) ppm; MS (ESI, MeOH): m/z (%) = 209.0 ([M − H]^−^, 100), 441.0 ([2M-H + Na]^−^, 3); analysis calcd for C_9_H_6_O_6_ (210.14): C 51.44, H 2.88; found: C 51.32, H 3.03.

### 4.7. [1,3]Dioxolo[4,5-f]benzofuran-6,7-dione (***11***)

A solution of **10** (4.25 g, 22.12 mmol) was suspended in dry DCM (200 mL) and at 0 °C oxalyl chloride (1.9 mL, 22.00 mmol) and dry DMF (200 µL) were added. The reaction mixture was stirred at r.t. for 14 h. The volatiles were removed under reduced pressure, the residue is re-dissolved in dry THF (50 mL) and evaporated to dryness. Column chromatography (SiO_2_, DCM) gave **11** (1.55 g, 36%) as slightly orange solid; m.p. 203–205 °C; R*_F_* = 0.69 (SiO_2_, DCM); IR (KBr): v = 3446br, 1818w, 1765m, 1716m, 1616s, 1480s, 1415m, 1299s, 1233s, 1176m, 1095m, 1037m cm^–1^; UV–vis (CHCl_3_): λ_max_ (log ε = 260 (3.82), 310 (3.56), 414 (3.33) nm; ^1^H-NMR (500 MHz, DMSO-d_6_): δ = 7.24 (s, 1H, 4-H), 7.14 (s, 1H, 7-H), 6.23 (s, 2H, 9-H) ppm; ^13^C-NMR (126 MHz, DMSO-d_6_): δ = 174.9 (C-2), 161.8 (C-8), 157.3 (C-1), 156.9 (C-6), 145.2 (C-5), 112.5 (C-3), 103.4 (C-9), 102.7 (C-4), 95.6 (C-7) ppm; MS (ESI, MeOH): *m/z* (%) = 225.0 ([M + H + MeOH]^+^, 68), 247.1 ([M + Na + MeOH]^+^, 100), 356.0 ([3(M + MeOH) + K + H]^2+^, 18), 467.7 ([4(M + MeOH) + K + H]^2+^, 30); analysis calcd for C_9_H_4_O_5_ (192.13): C 56.26, H 2.10; found: C 55.97, H 2.27.

### 4.8. 1-(2,4,5-Trimethoxyphenyl)ethan-1-one-1-^13^C (***12***)

To a solution of 1,2,4-trimethoxybenzene (3, 2.0 g, 11.9 mmol) in DCM (50 mL) at −20 °C TiCl_4_ (1.44 mL, 13.1 mmol) was added followed by adding dropwise ^13^C-acetylchloride (0.93 mL, 13 mmol). The resulting mixture was stirred for 2 h, poured into 2M hydrochloric acid and extracted with chloroform (3 × 50 mL). The combined organic extracts were washed with brine and dried (MgSO_4_). Evaporation followed by column chromatography gave **12** (2.4 g, 95%) as a pale yellow crystalline solid.; m.p. 189–192 °C; R*_F_* = 0.25 (SiO_2_, hexanes/ethyl acetate, 1:1); ^1^H-NMR (400 MHz, CDCl_3_): δ = 7.42 (d, *J* = 4.2 Hz, 1H, 6-H), 6.50 (d, *J* = 1.6 Hz, 1H, 3-H), 3.94 (s, 3H, OMe), 3.91 (s, 3H, OMe), 3.87 (s, 3H, OMe), 2.59 (d, *J* = 6.2 Hz, 3H, 2′-H) ppm; ^13^C-NMR (101 MHz, CDCl_3_): δ = 197.2 (C-1′), 155.6 (d, *J* = 2.2 Hz, C-4), 153.9 (C-2), 143.0 (d, *J* = 4.0 Hz, C-5), 119.2 (d, *J* = 54.5 Hz, C-1), 112.54 (d, *J* = 1.5 Hz, C-6), 96.42 (d, *J* = 3.4 Hz, C-3), 56.3 (OMe), 56.1 (OMe), 56.1 (OMe), 32.03 (d, *J* = 42.7 Hz, C-2′); MS (ESI): *m/z* (%) = 212.37 ([M + H]^+^, 100).

### 4.9. 2-Oxo-2-(2,4,5-trimethoxyphenyl)acetic-2-^13^C Acid (***13***)

A suspension of **12** (2.0 g, 9.47 mmol) and selenium dioxide (2.0 g, 18 mmol) in pyridine (20 mL) was stirred for 4 h at 80 °C. Work-up as described above followed by re-crystallization from EtOAc gave **13** (1.20 g, 53%) as yellow solid; m.p. 163–165 °C; R*_F_* = 0.35 (SiO_2_, CHCl_3_/MeOH, 2:1); ^1^H-NMR (400 MHz, DMSO-d_6_): δ = 7.22 (d, *J* = 4.2 Hz, 1H, 6-H), 6.82 (d, *J* = 1.8 Hz, 1H, 3-H), 3.92 (s, 3H, OMe), 3.84 (s, 3H, OMe), 3.76 (s, 3H, OMe) ppm; ^13^C-NMR (101 MHz, DMSO-d_6_): δ = 186.5 (C-2′), 168.1 (d, *J* = 74.3 Hz, C-1′), 157.7 (d, *J* = 2.0 Hz, C-2), 156.9 (d, *J* = 0.8 Hz, C-4), 144.2 (d, *J* = 4.3 Hz, C-5), 113.6 (d, *J* = 59.5 Hz, C-1), 110.6 (d, *J* = 1.7 Hz, C-6), 98.6 (d, *J* = 3.0 Hz, C-3), 57.6 (OMe), 56.7 (OMe), 56.3 (OMe) ppm; MS (ESI, MeOH): *m/z* (%) = 239.8 ([M − H]^−^, 100).

### 4.10. 6-Hydroxy-5-methoxy-3,3-bis(2,4,5-trimethoxyphenyl)benzofuran-2(3H)-one-3-^13^C (***15***)

A suspension of **13** (200 mg, 0.83 mmol) in conc. hydrochloric acid (25 mL) was stirred 0 °C for 2 h. The reaction mixture was neutralized with an aqueous sat. soln. of NaHCO_3_ and extracted with chloroform (3 × 25 mL). The organic extracts were dried (MgSO_4_), the solvent was removed under diminished pressure and the remaining residue was subjected to column chromatography (SiO_2_, CHCl_3_/MeOH, 9:1) to afford **15** as a slightly yellow solid (13 mg, 3%); m.p. 103–105 °C; R*_F_* = 0.66 (SiO_2_, CHCl_3_/MeOH, 9:1); ^1^H-NMR (500 MHz, toluene-d_8_): δ = 7.17–6.83 (m, 2H, 2 × 6′-H), 7.01 (s, 1H, 7-H), 6.70 (d, *J* = 2.6 Hz, 1H, 4-H 6.28 (s, 2H, 2 × 2′-H), 3.49 – 3.38 (m, 6H, 2 × OMe(2′)), 3.40 (s, 6H, 2 × OMe(5′)), 3.30 (s, 6H, 2 × OMe(4′)), 3.06 (s, 3H, OMe(5)) ppm; ^13^C-NMR (126 MHz, toluene-*d_8_*): δ = 175.91 (d, *J* = 52.5 Hz, C-2), 148.13 (C-2′), 146.40 (C-4′), 142.8 (C-5), 142.8(C-5′), 137.10 (C-7a), 124.4 (d, J = 57.3 Hz, C-1′), 122.72 (d, *J* = 45.6 Hz, C-3b), 108.80 (d, *J* = 1.7 Hz, C-4), 99.8 (C-3′), 97.80 (C-7), 57.41 (C-3), 56.58 (OMe(2′)), 55.65 (OMe(5′)), 55.55 (OMe(4′)), 55.34 (OMe(5)) ppm; MS (ESI, MeOH): *m/z* (%) = 512.1 ([M − H]^−^, 100), 536.1 ([M + Na]^+^, 100), 782.0 ([3M + Na + H]^2+^, 33), 791.1 ([3M + K + H]^2+^, 26).

### 4.11. 6-Hydroxy-5-methoxy-3-(2,4,5-trimethoxyphenyl)benzofuran-2(3H)-one (***17***)

Compound **6** (250 mg, 0.73 mmol) was suspended in hydrochloric acid (25 mL) and zinc (250 mg, 3.82 mmol) was added at 0 °C. The reaction was stirred for 4 h at room temperature, diluted with water (20 mL) and extracted with EtOAc (3 × 50mL). The combined organic extracts were washed with brine and dried (MgSO_4_). Purification by column chromatography (SiO_2_, hexanes/CHCl_3_, 1:1) gave **17** (112 mg, 43%) as slightly yellow solid; m.p. 143–147 °C; R*_F_* = 0.32 (SiO_2_, hexanes/CHCl_3_, 1:1); ^1^H-NMR (500 MHz, CDCl_3_): δ = 6.78 (s, 1H, 7-H), 6.68 (s, 1H, 6′-H), 6.56–6.53 (m, 2H, 4-H + 3′-H), 5.78 (s, 1H, OH), 4.86 (s, 1H, 3-H3), 3.88 (s, 3H, OMe(4′)), 3.82 (s, 3H, OMe(2′)), 3.79 (s, 3H, OMe(5)), 3.70 (s, 3H, OMe(5′)) ppm; ^13^C-NMR (126 MHz, CDCl_3_): δ = 176.57 (C-2), 151.72 (C-2′), 149.96 (C-4′), 148.03 (C-7a), 146.24 (C-6), 143.61 (C-5), 143.36 (C-5′), 118.20 (C-3a), 116.33(C-1′), 114.11 (C-6‘), 106.85 (C-4), 98.63 (C-3‘), 98.44 (C-7), 56.83 (OMe(5′)), 56.79 (OMe(2′)), 56.62 (OMe(5)), 56.18 (OMe(4′)), 46.19 (C-3) ppm; MS (ESI, MeOH): *m/z* (%) = 345.6 ([M − H]^−^, 100); analysis calcd for C_18_H_18_O_7_ (346.33): C 62.44, H 5.24; found: C 62.26, H 4.98.

### 4.12. 5-Methoxy-3-(2-methoxyphenyl)benzofuran-2,6-dione (***18***)

Compound **2** (240 mg, 1.0 mmol) and anisole (440 mg, 4 mmol) were suspended in hydrochloric acid (25 mL) and the mixture was stirred for 4 h at 40 °C, diluted with water (10 mL), extracted with CHCl_3_ (3 × 25 mL). The combined organic extracts were washed with brine, dried (MgSO_4_), the solvent was evaporated and the residue re-crystallized from EtOAc to yield **18** (211 mg, 74%) as a red crystalline solid; m.p. 194–196 °C; R*_F_* = 0.54 (SiO_2_, CHCl_3_); ^1^H-NMR (500 MHz, CDCl_3_): δ = 7.52–7.45 (m, 2H, 6′-H + 4‘-H), 7.11 (td, *J* = 7.6, 1.0 Hz, 1H, 5′-H), 7.05 (dd, *J* = 8.3, 1.0 Hz, 1H, 3′-H), 6.27 (s, 1H, 4-H), 6.09 (s, 1H, 7-H), 3.89 (s, 3H, OMe(2′)), 3.82 (s, 3H, OMe(5)) ppm; ^13^C-NMR (126 MHz CDCl_3_): δ = 181.29 (C6), 160.53 (C2), 157.38 (C2′), 154.42 (C5), 140.69 (C7a), 132.40 (C4‘), 131.67 (C6‘), 126.18 (C3a), 121.29 (C5‘), 117.65 (C1′), 111.88 (C3‘), 104.49 (C7), 99.83 (C4), 56.41 (OMe(2′)), 55.77 (OMe(5′)) ppm; MS (ESI, MeOH): *m/z* (%) = 285.4 ([M + H]^+^, 100), 307.3 ([M + Na]^+^, 38); analysis calcd for C_16_H_12_O_5_ (284.26): C 67.60, H 4.26; found: C 67.41, H 4.39.

### 4.13. 3-(4-Aminophenyl)-5-methoxybenzofuran-2,6-dione (***19***)

To a suspension of **2** (500 mg, 2.08 mmol) in hydrochloric acid (25 mL) Boc-aniline (781 mg, 4 mmol) was added and the mixture was stirred for 4 h at 40 °C. Usual aqueous workup followed by column chromatography (SiO_2_, CHCl_3_/MeOH, 95:5) and recrystallization from EtOAc gave **19** (243 mg, 43%) as black crystalline solid; m.p. 243–246 °C; R*_F_* = 0.14 (SiO_2_, CHCl_3_); IR (ATR): ν = 3456w, 3331m, 3219w, 1780m, 1662m, 1643m, 1628m, 1600s, 1575s, 1551s, 1515s, 1506m, 1470w, 1445m, 1439m, 1415s, 1355m, 1331m, 1227s, 1181s, 1157s, 995m, 945m, 850m, 840s, 832s, 821m, 789m, 773m, 687m, 679m, 602m cm^−1^; UV–Vis (MeOH): λ_max_ (log ε) 251 (3.67), 317 (3.80), 507 (3.92) nm; ^1^H-NMR (500 MHz, DMSO-d_6_): δ = 7.71 (d, *J* = 8.7 Hz, 2H, 2′-H), 6.75 (s, 1H, 4-H), 6.72 (d, *J* = 8.8 Hz, 1H, 3′-H), 6.26 (s, 2H, NH), 6.12 (s, 1H, 7-H), 3.85 (s, 3H, OMe) ppm; ^13^C NMR (126 MHz, DMSO-d_6_): δ = 179.71 (C-6), 167.28 (C-2), 159.86 (C-7a), 153.98 (C-5), 152.18(C-3), 131.58 (C-2′), 130.82 (C-3a), 126.93 (C-1′), 116.23 (C-4′), 114.06 (C-3′), 103.10 (C-7), 99.84 (C-4), 56.03 (OMe) ppm; MS (ESI, MeOH): *m/z* (%) = 270.2 ([M + H]^+^, 100), 292.0 ([M + Na]^+^, 14), 302.1 ([M + H + MeOH]^+^, 20), 423.5 ([3M + Ca]^2+^, 18), 560.8 ([2M + Na]^+^, 90); analysis calcd for C_15_H_11_NO_4_ (269.25): C 66.91, H 4.12, N 5.20; found: C 66.72, H 4.34, N 5.02.

### 4.14. N-(4-(5-Methoxy-2,6-dioxo-2,6-dihydrobenzofuran-3-yl)phenyl)benzamide (***20***)

To a solution of **19** (200 mg, 0.74 mmol) in DCM (50 mL) and triethylamine (0.11 mL, 0.8 mmol) at 0 °C benzoylchloride (0.93 mL, 0.8 mmol) was added and the reaction was stirred for 6 h. Usual aqueous workup followed by column chromatography (SiO_2_, CHCl_3_/MeOH, 9:1) gave **20** (191 mg, 69%) as a red solid; m.p. 259–264 °C; R*_F_* = 0.11 (SiO_2_, CHCl_3_); IR (ATR): ν = 3377w, 3021w, 2973w, 2934w, 1811w, 1782m, 1749w, 1669m, 1629s, 1587s, 1572s, 1512s, 1489s, 1452m, 1421m, 1408s, 1351m, 1321s, 1303m, 1250s, 1232s, 1181s, 1158s, 1124m, 1105m, 1079w, 1066w, 1020w, 991m, 934m, 904m, 854m, 847s, 837s, 827s, 799m, 789m, 720s, 699m, 690m, 669m, 625s, 610s, 546s cm^−1^; UV–Vis (methanol): λ_max_ (log ε) = 229 (3.89), 307 (3.97), 452 (3.92) nm; ^1^H-NMR (500 MHz, pyridine-d_5_): δ = 11.36 (s, 1H, NH), 8.37–8.32 (m, 2H, 2 × 2′-H), 8.27–8.21 (m, 2H, 2 × 2′’-H), 8.09–8.05 (m, 2H, 2 × 3′-H), 7.50–7.46 (m, 1H, 4′’-H), 7.45–7.38 (m, 2H, 2 × 3′’-H), 6.71 (s, 1H, 4-H), 6.32 (s, 1H, 7-H), 3.77 (s, 3H, OMe) ppm; ^13^C-NMR (126 MHz, pyridine-d_5_): δ = 180.98 (C-6), 167.90 (C=O), 167.75 (C-2), 160.91(C-7a), 156.36 (C-5), 143.12 (C-4′), 138.18 (C-3a), 136.42 (C-1′’), 132.47 (C-4′’), 131.38 (2x C-2′), 129.19 (2x C-3′’), 128.86 (2 × C-2′’), 127.09 (C-3), 125.27 (C-1′), 121.63 (2 × C-3′), 105.16 (C-7), 99.25 (C-4), 56.70 (OMe) ppm; MS (ESI, MeOH): *m/z* (%) = 374.20 ([M + H]^+^, 46), 396.07 ([M + Na]^+^, 18), 406.0 ([M + MeOH + H]^+^, 100), 428.1 ([M + MeOH + Na]^+^, 32), 766.7 ([2M + Na]^+^, 55), 800.7 ([2M + MeOH + Na]^+^, 70), 832.9 ([2M + 2MeOH + Na]^+^, 64); analysis calcd for C_22_H_15_NO_5_ (373.36): C 70.77, H 4.05, N 3.75; found: C 70.51, H 4.35, N 3.52.

### 4.15. 6-Hydroxy-5-methoxy-3-phenyl-3-(2,4,5-trimethoxyphenyl)benzofuran-2(3H)-one (***21***)

To a solution of **2** (240 mg, 1.0 mmol) and benzene (45 µL, 0.5 mmol) hydrochloric acid (25 mL) was added and the mixture was stirred for 2 h. Usual aqueous work-up followed by column chromatography (SiO_2_, hexanes/CHCl_3_, 1:1) gave **21** (74 mg, 18%) as a pale yellow crystalline solid; m.p. 112–117 °C; R*_F_* = 0.63 (SiO_2_, CHCl_3_); IR (KBr): ν = 3432br, 3081w, 3062w, 3008w, 2961w, 2941w, 2842w, 1763w, 1638s, 1586s, 1527m, 1516m, 1461m, 1427w, 1382w, 1412m, 1388m, 1348s, 1322w, 1233s, 1190s, 1140m, 1043m, 991m, 936m, 852s, 824m, 781m, 689m cm^−1^; UV-Vis (MeOH): λ_max_ (log ε) = 239 (3.05), 284 (3.01), 331 (3.37) nm^1^H NMR (500 MHz, CDCl_3_): δ = 7.45 (d, *J* = 8.0 Hz, 2H, 2′’H_a_ + 2′’-H_b_), 7.38–7.31 (m, 3H, 3”-H_a_ + 3′’-H_b_ + 4′’-H), 6.81 (s, 1H, 7-H), 6.54 (s, 1H, 4-H), 6.50 (s, 1H, 3′-H), 6.41 (s, 1H, 6′-H), 5.85 (s, 1H, OH), 3.86 (s, 3H, OMe(2′)), 3.81 (s, 3H, OMe(5)), 3.62 (s, 3H, OMe(5′)), 3.61 (s, 3H, OMe(4′)) ppm; ^13^C NMR (126 MHz, CDCl_3_): δ = 178.27 (C-2), 151.58 (C-2′), 149.83 (C-4), 147.79 (C-7a), 146.46 (C-6), 143.63 (C-5), 143.13 (C-5′), 138.09 (C-1′’), 129.27 (2 × C-2′’), 128.53 (2 × C-3′’), 128.32 (C-4′’), 123.36 (C-1′), 120.65 (C-3b), 114.05 (C-6′), 108.18 (C-4), 99.07 (C-3′), 98.57 (C-7), 57.96 (C-3), 56.9 (OMe(4′)), 56.9 (OMe(5′)), 56.8 (OMe(5)), 56.2 (OMe(2′)) ppm; MS (ESI): *m/z* (%) = 421.2 ([M − H]^−^, 100); analysis calcd for C_24_H_22_O_7_ (422.43): C 68.24, H 5.25; found: C 68.10, H 5.37.

## 5. Conclusions

The reaction **2** in the presence of hydrochloric acid led to the formation of the highly substituted *p*-QM **6**. The putative mechanism of this reaction was deduced from ^13^C-NMR labeling experiments as well by the ESI-MS identification of several intermediates. The mechanism for the formation of **6** includes the equilibrium between a Friedel–Crafts alkylation and a retro Friedel–Crafts alkylation.

## Supplementary Materials

The following are available online: NMR spectra of compounds (^1^H and ^13^C (APT)-NMR).
