# Supplementary material for: In the Mists of a Fungal Metabolite: An Unexpected Reaction of 2,4,5-Trimethoxyphenylglyoxylic Acid
Source: Molecules. 2020 Apr 23;25(8):1978. doi: 10.3390/molecules25081978 (PMC7221594; doi:10.3390/molecules25081978)
Supplement: Supplementary file 1 [file molecules-25-01978-s001.pdf]

molecules

Supplementary material

In the mists of a fungal metabolite: An unexpected reaction of 2,4,5-trimethoxyphenylglyoxylic acid

Immo Serbian [1], Anne Loesche [1], Sven Sommerwerk [1,] Phil Liebing [2], Dieter Ströhl [1], René Csuk [1,*]

[1] Martin-Luther-Universität Halle-Wittenberg, Organische Chemie, Kurt-Mothes Str. 2, D-06120 Halle (Saale), Germany

[2] Otto von Guericke Universität Magdeburg, Chemisches Institut, Universitätsplatz 2, D-39106 Magdeburg, Germany

Temperature dependent NMR of **15** and solvent exchange ($CDCl_3$, toluene-d_8, dmso-d_6)

$CDCl_3$: 40 °C

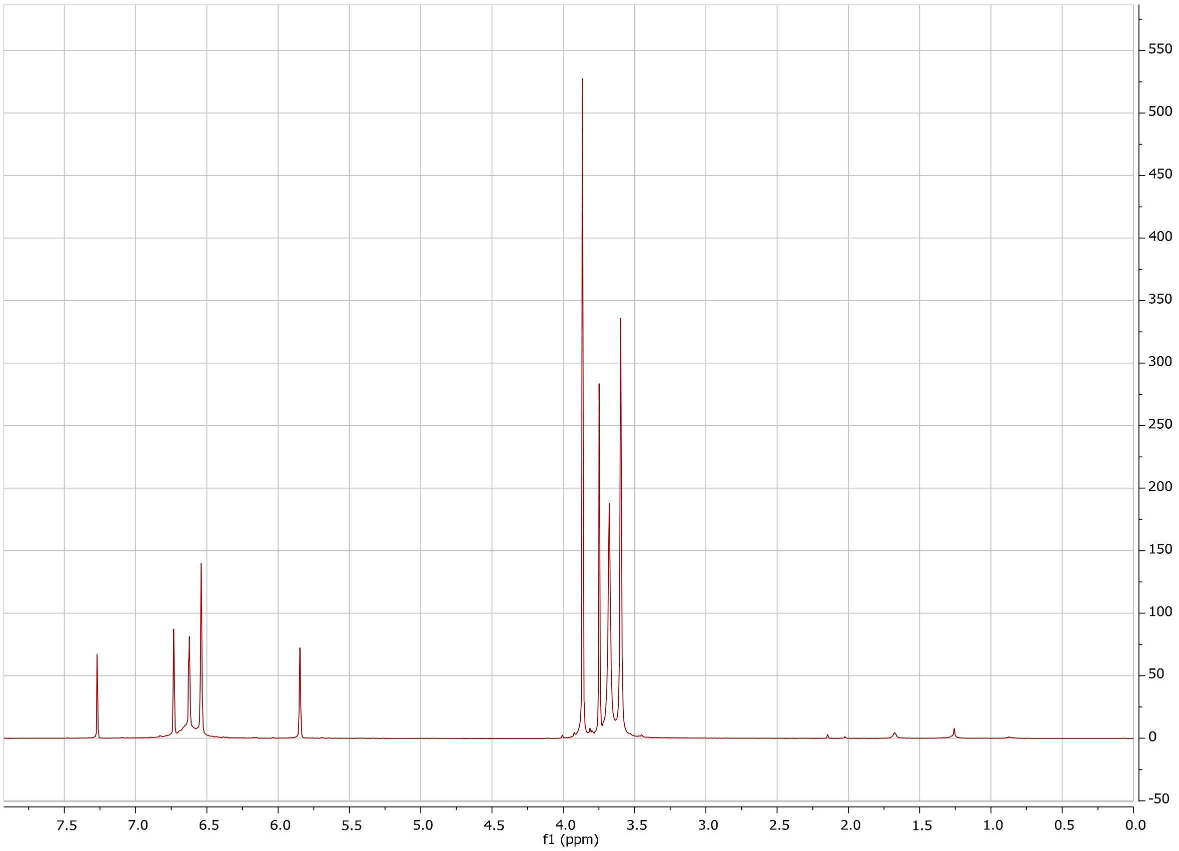

$CDCl_3$: 27 °C

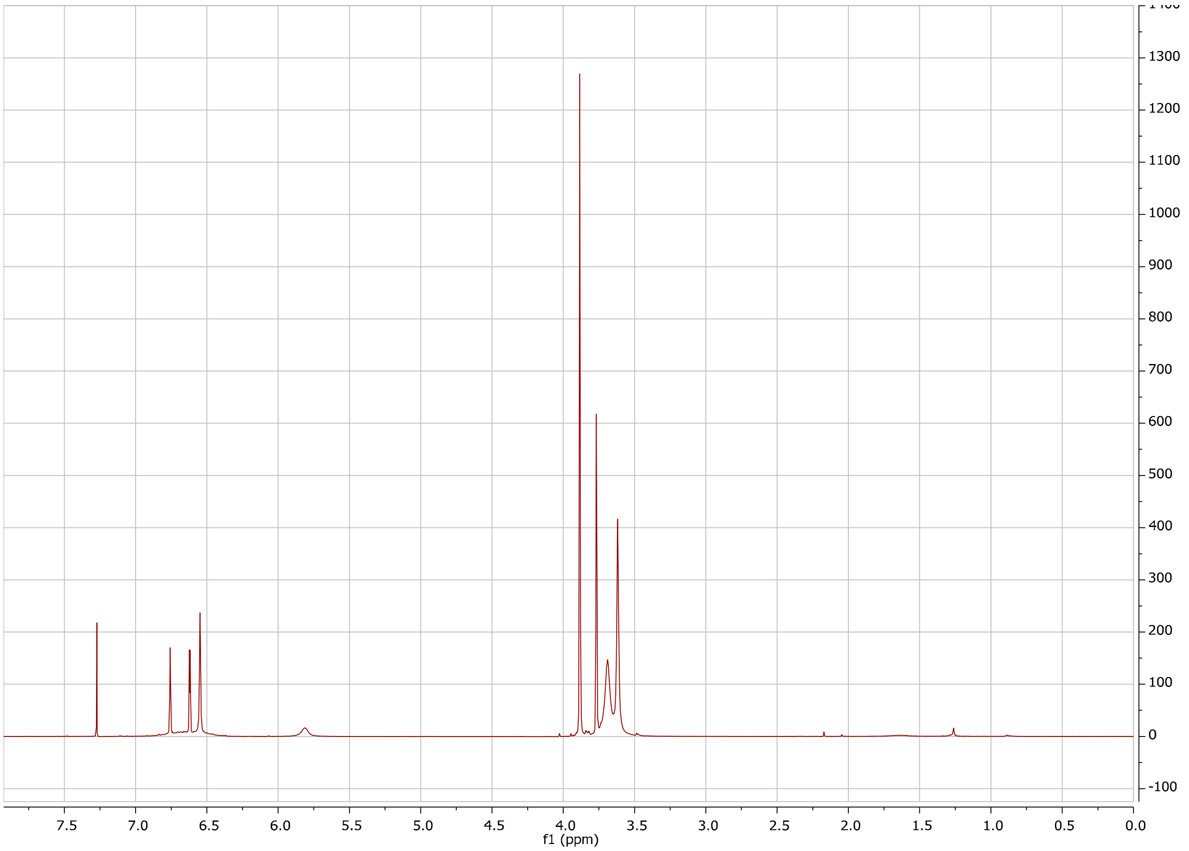

CDCl₃: 10 °C

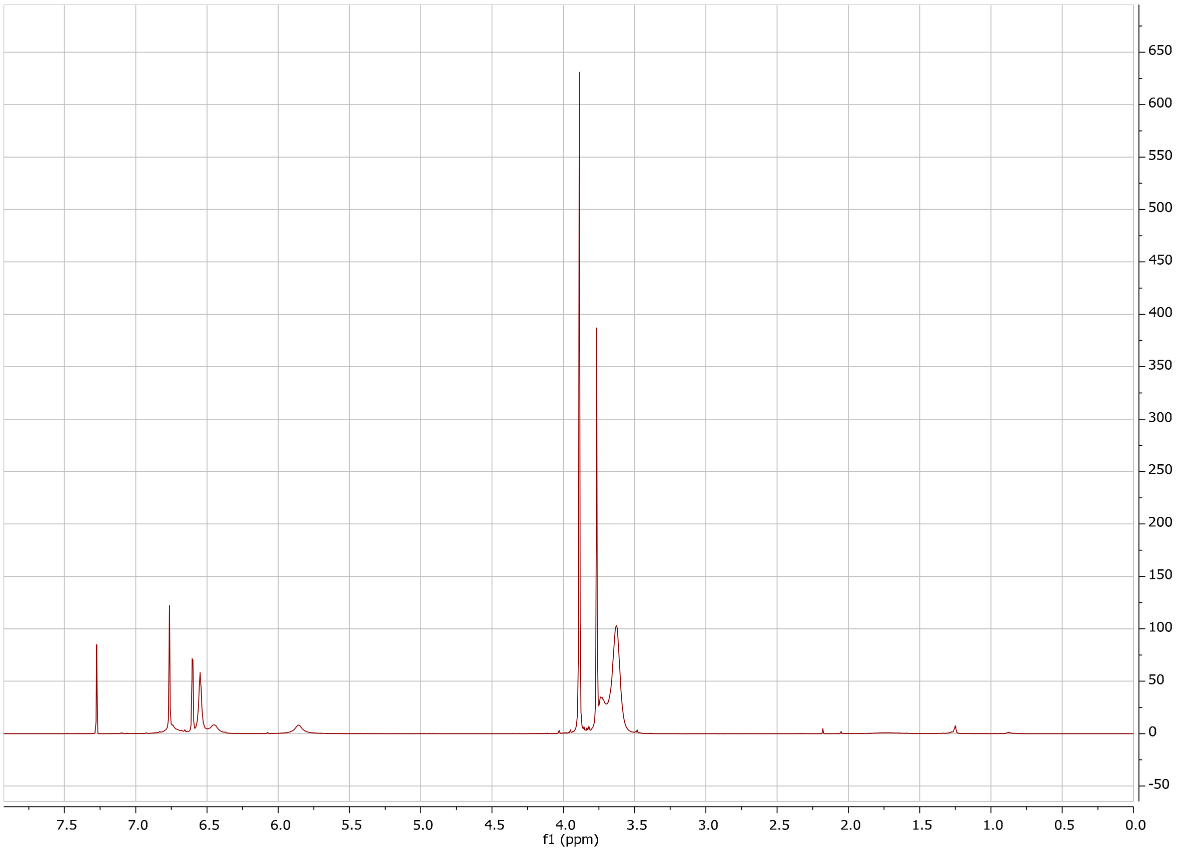

CDCl₃: 0 °C

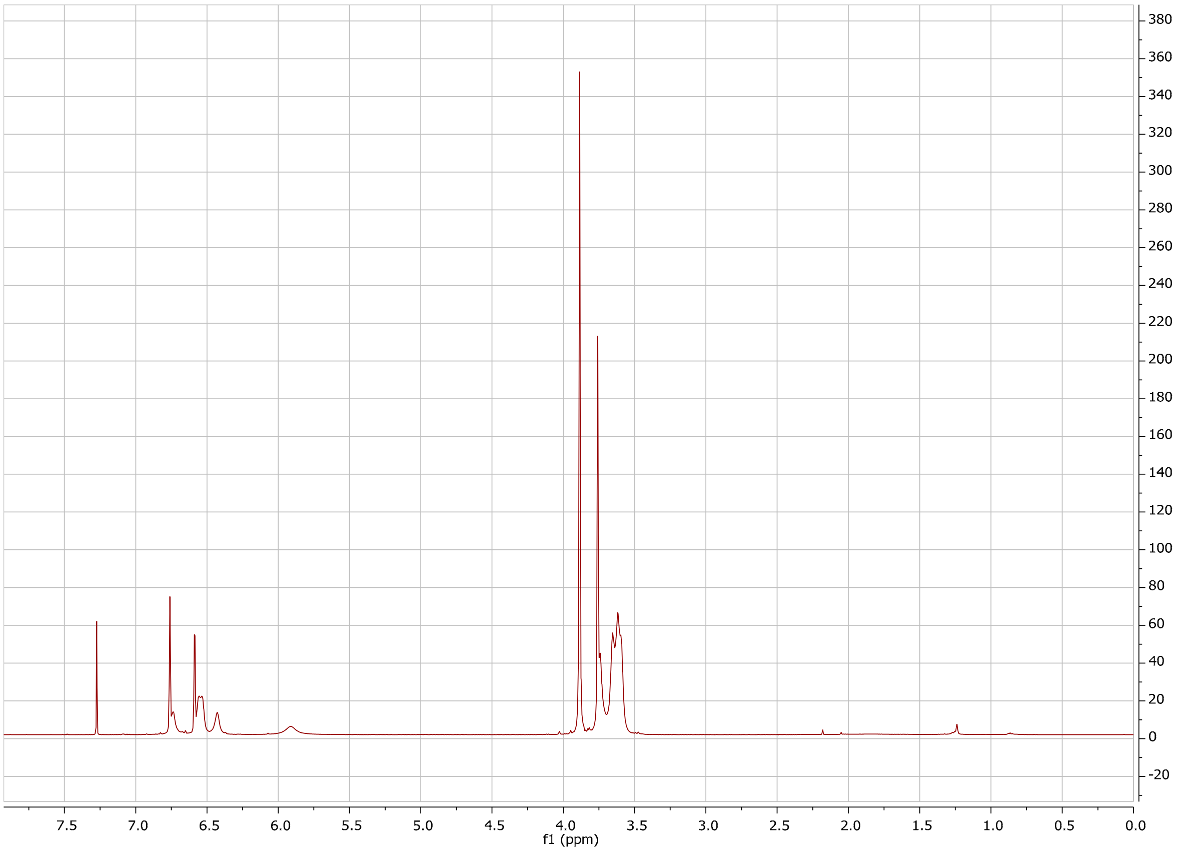

$CDCl_3$: -10 °C

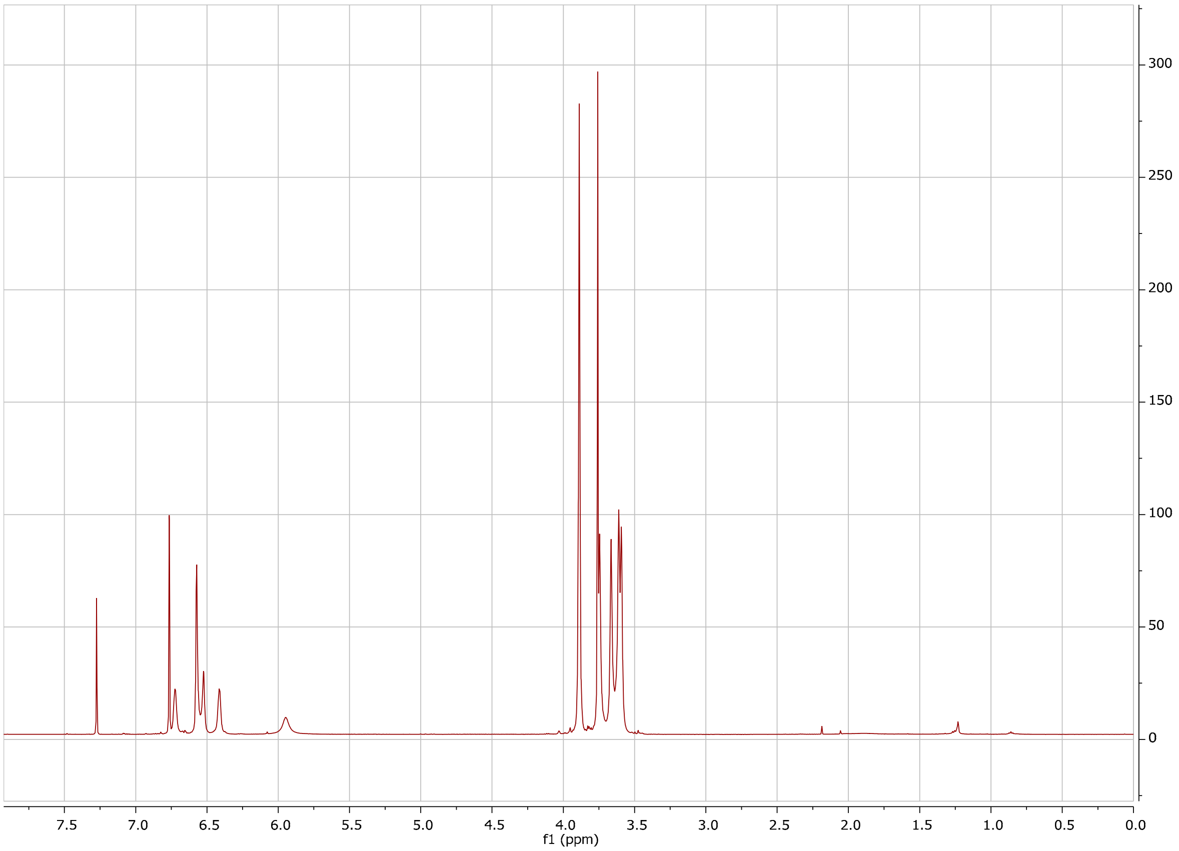

$CDCl_3$: -20 °C

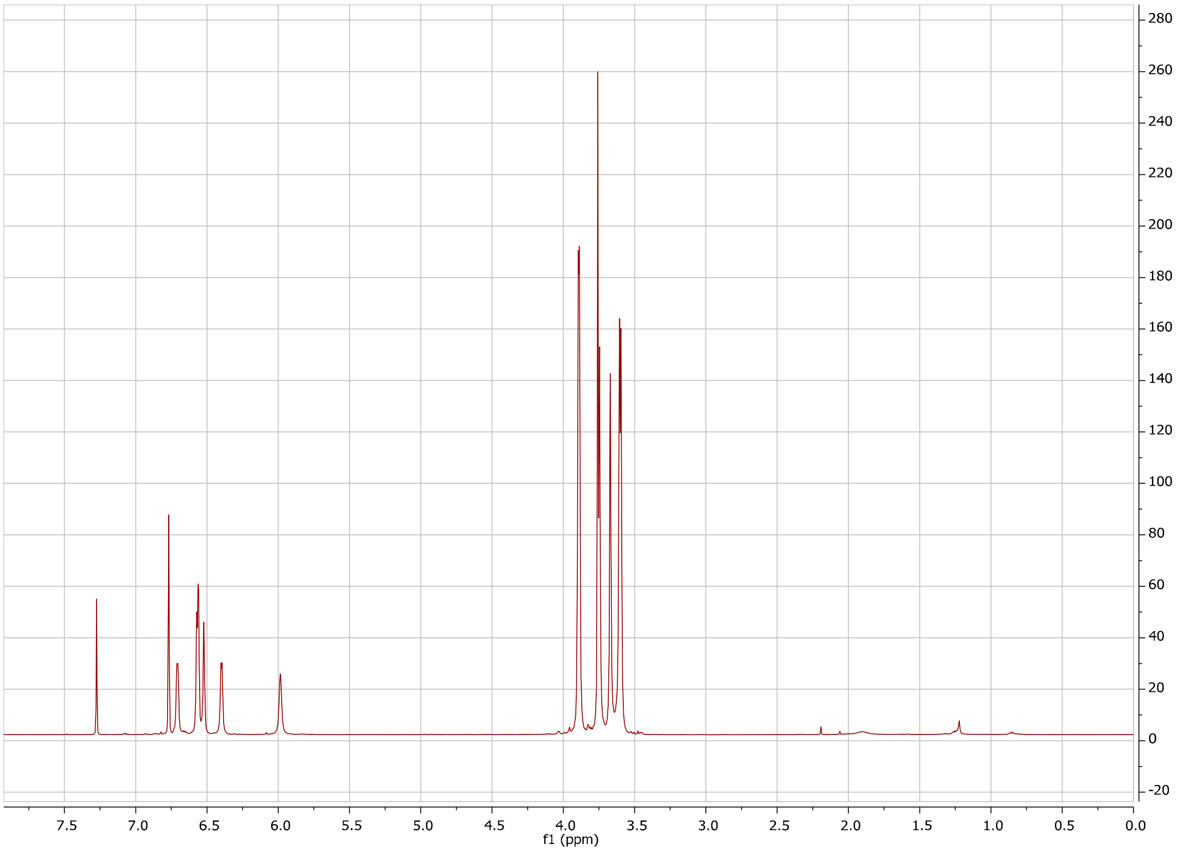

CDCl3: -30 °C

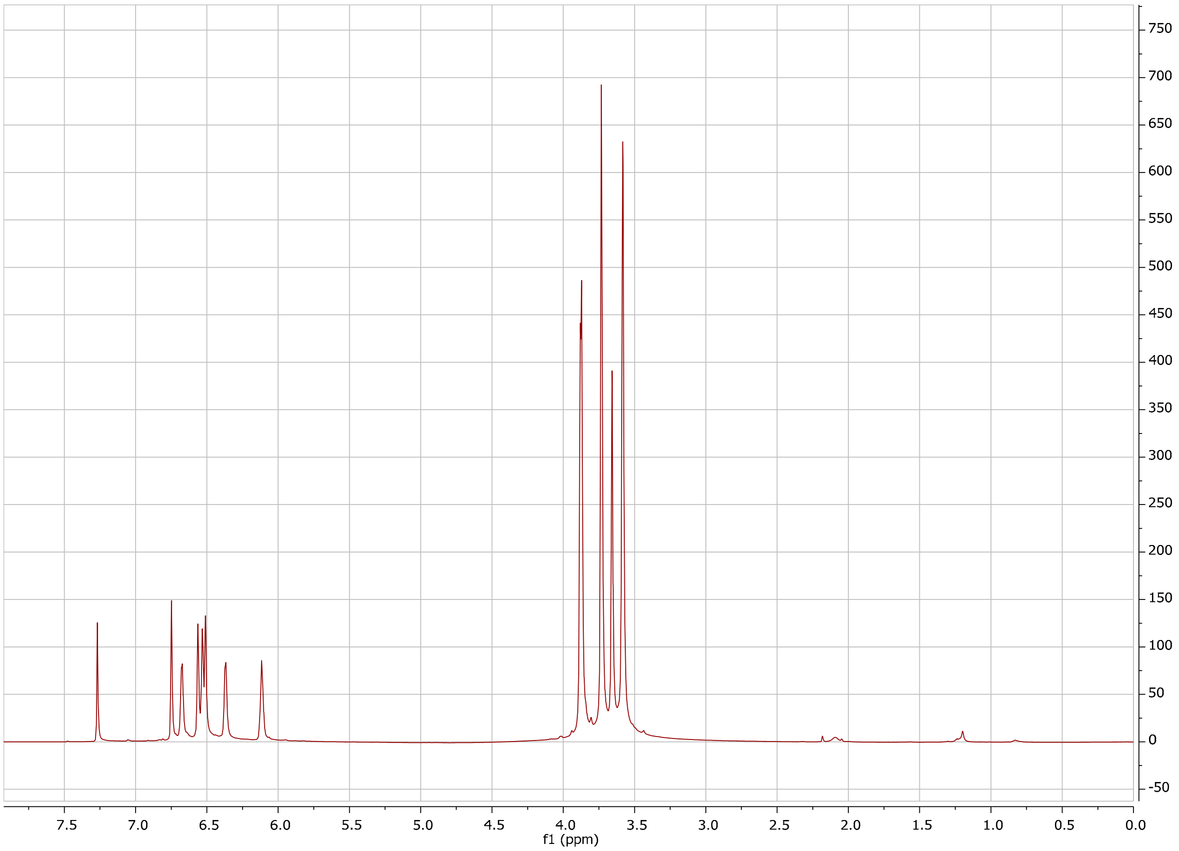

CDCl3: -50 °C

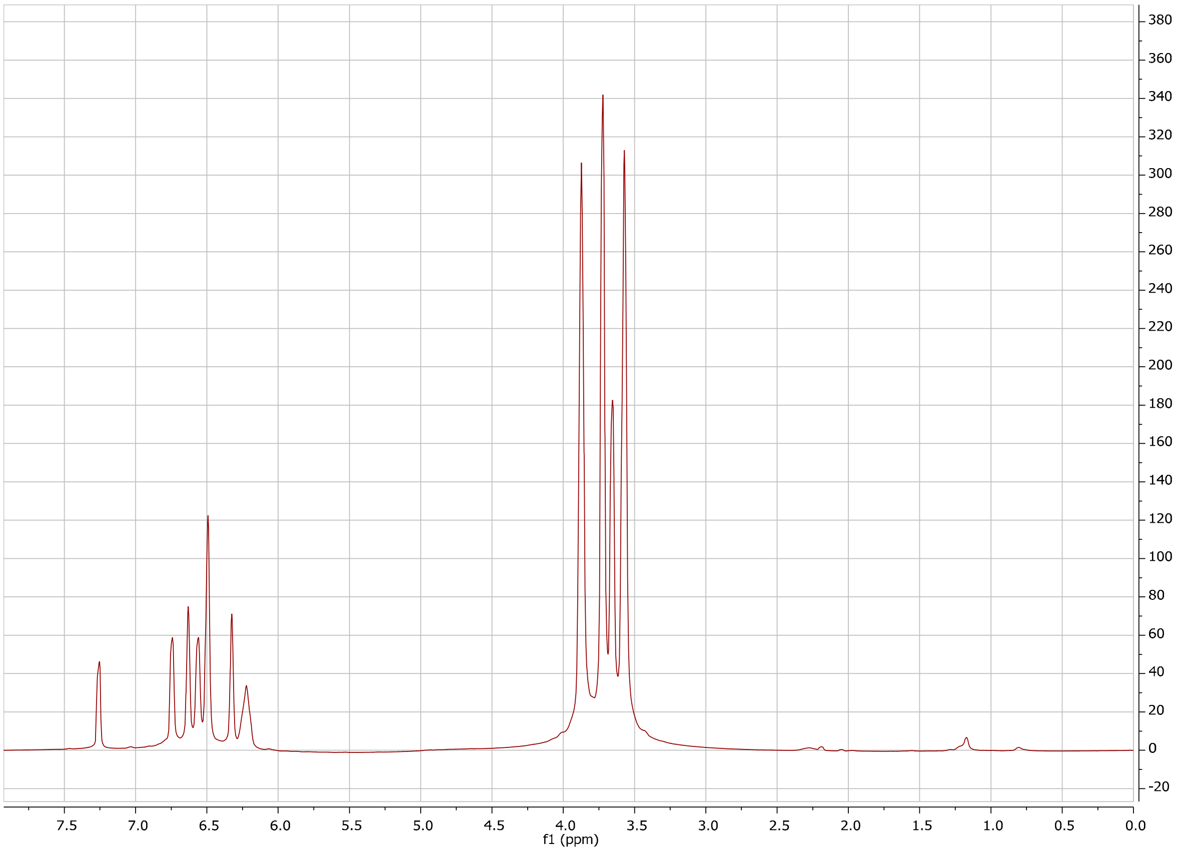

DMSO-d_6: 27 °C

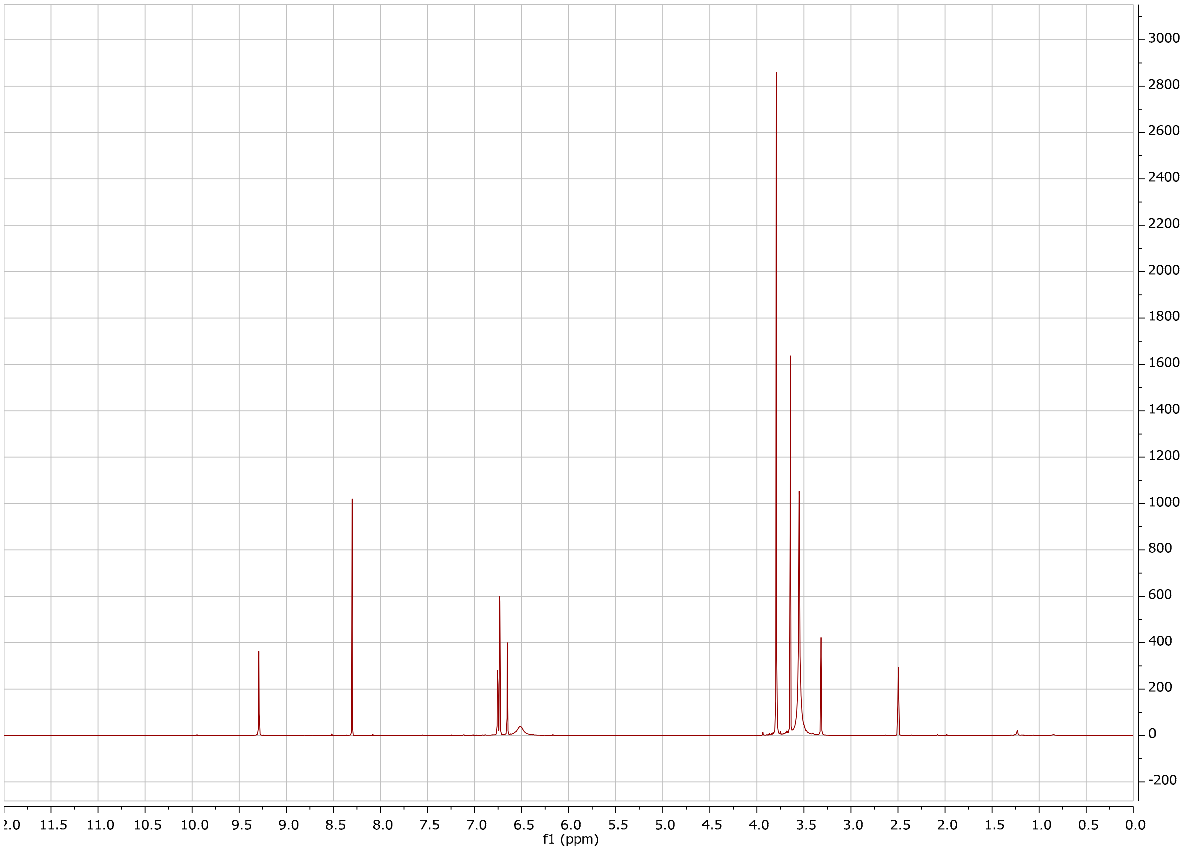

Toluene-d_8: 27 °C

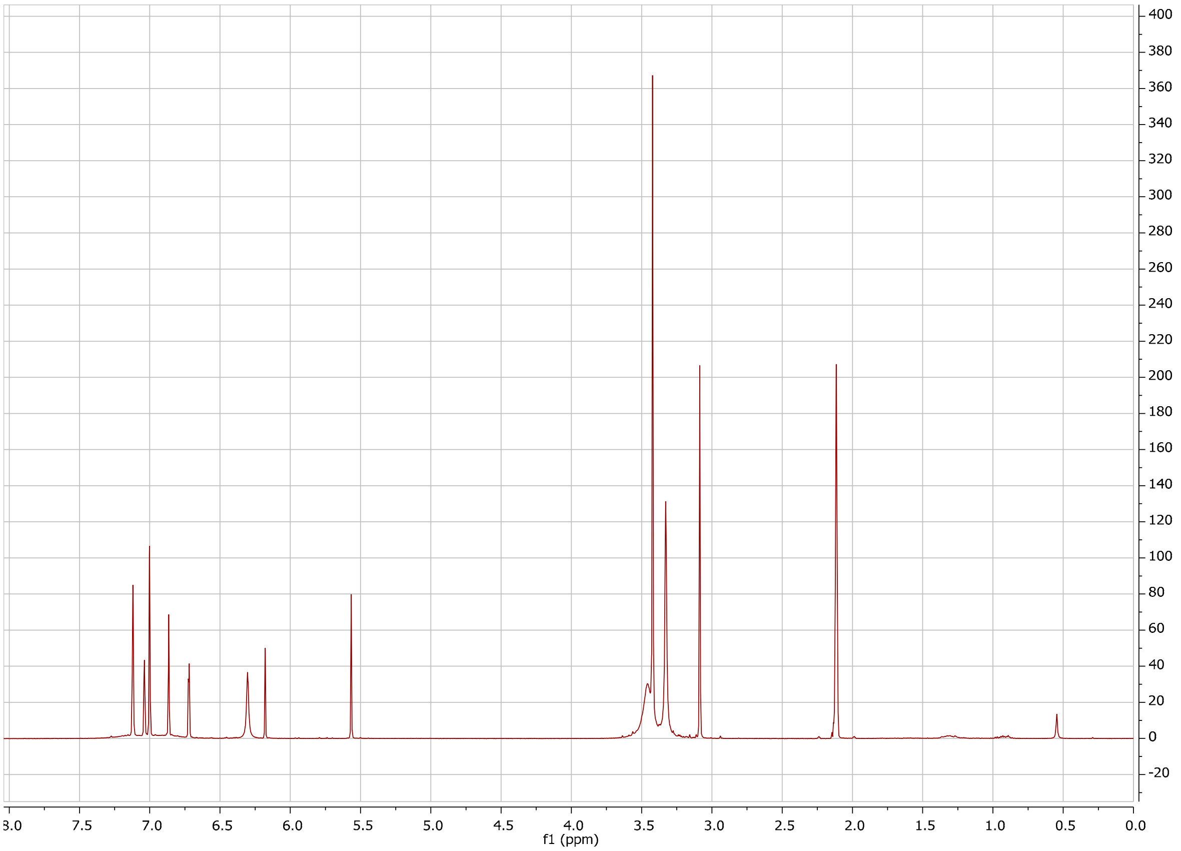
f1 (ppm)

^{1}H and ^{13}C (APT) NMR spectra

4

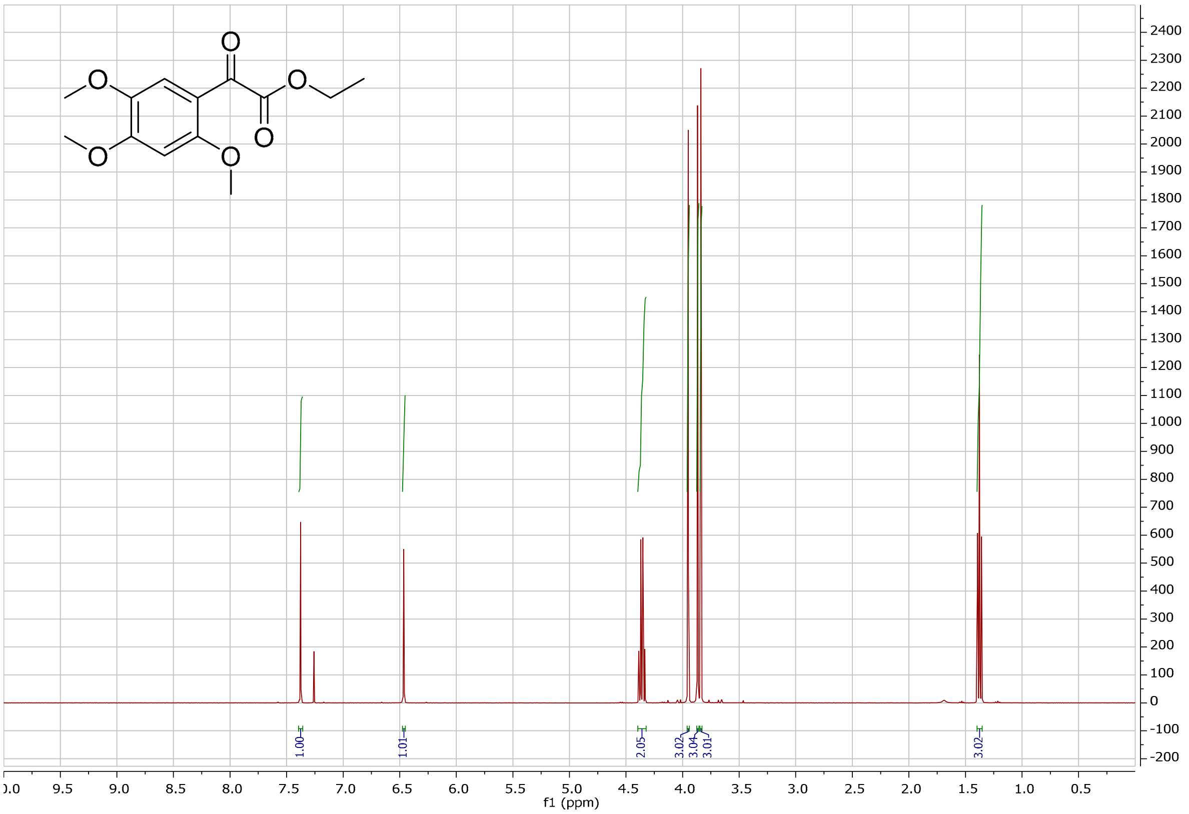

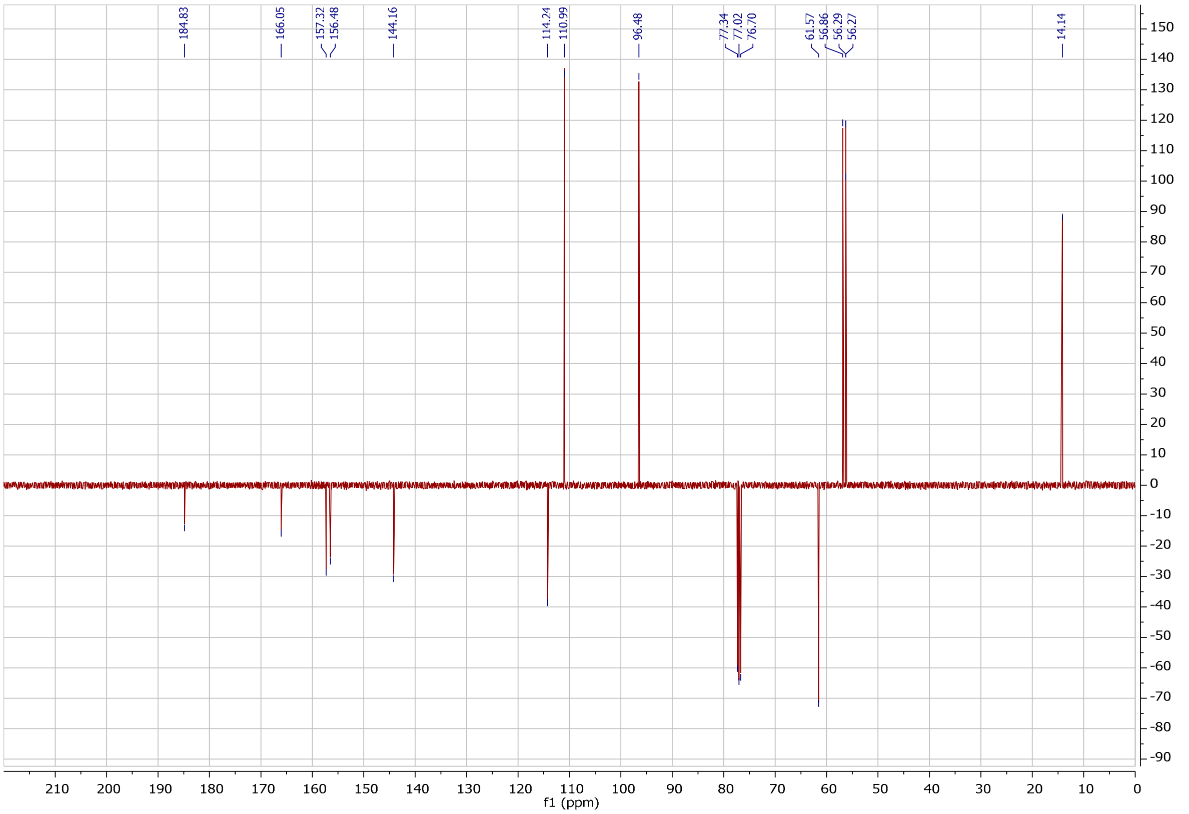

5

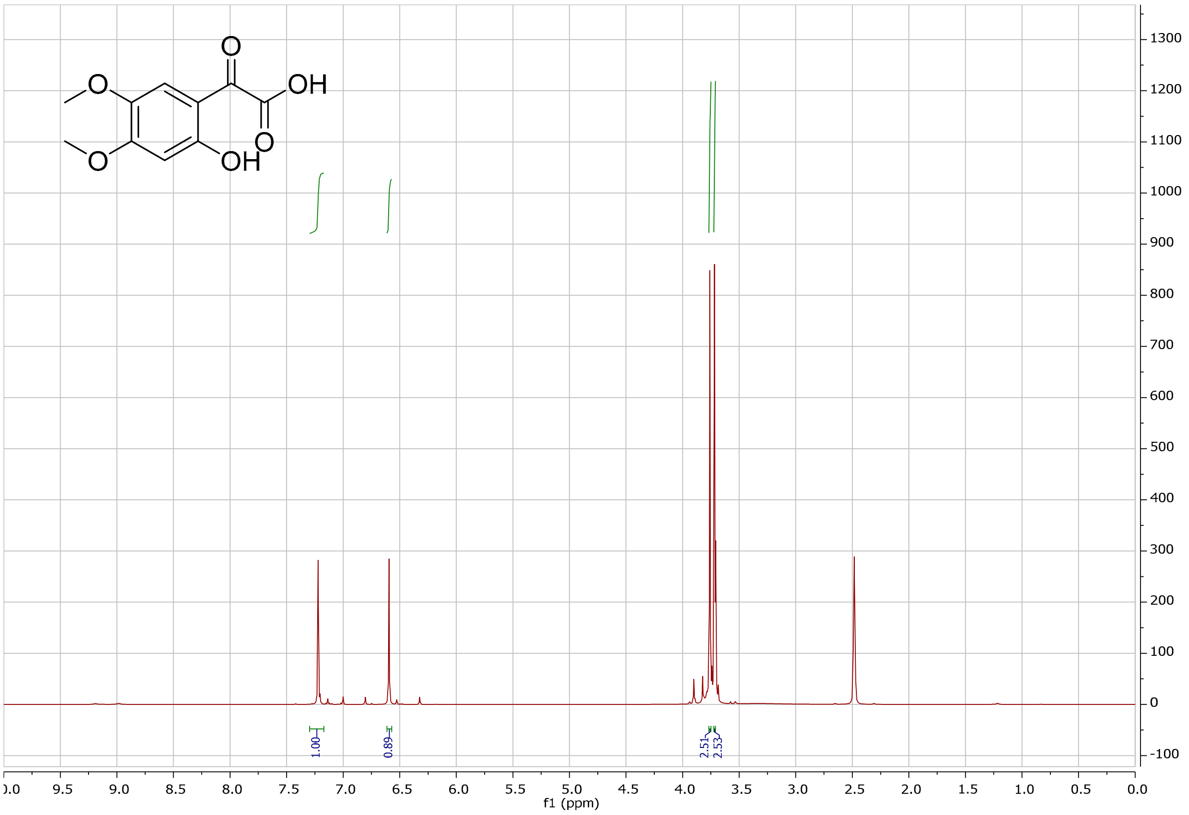
O
OH
O
O
O
OH
1.00
0.89
2.51
2.53
f1 (ppm)

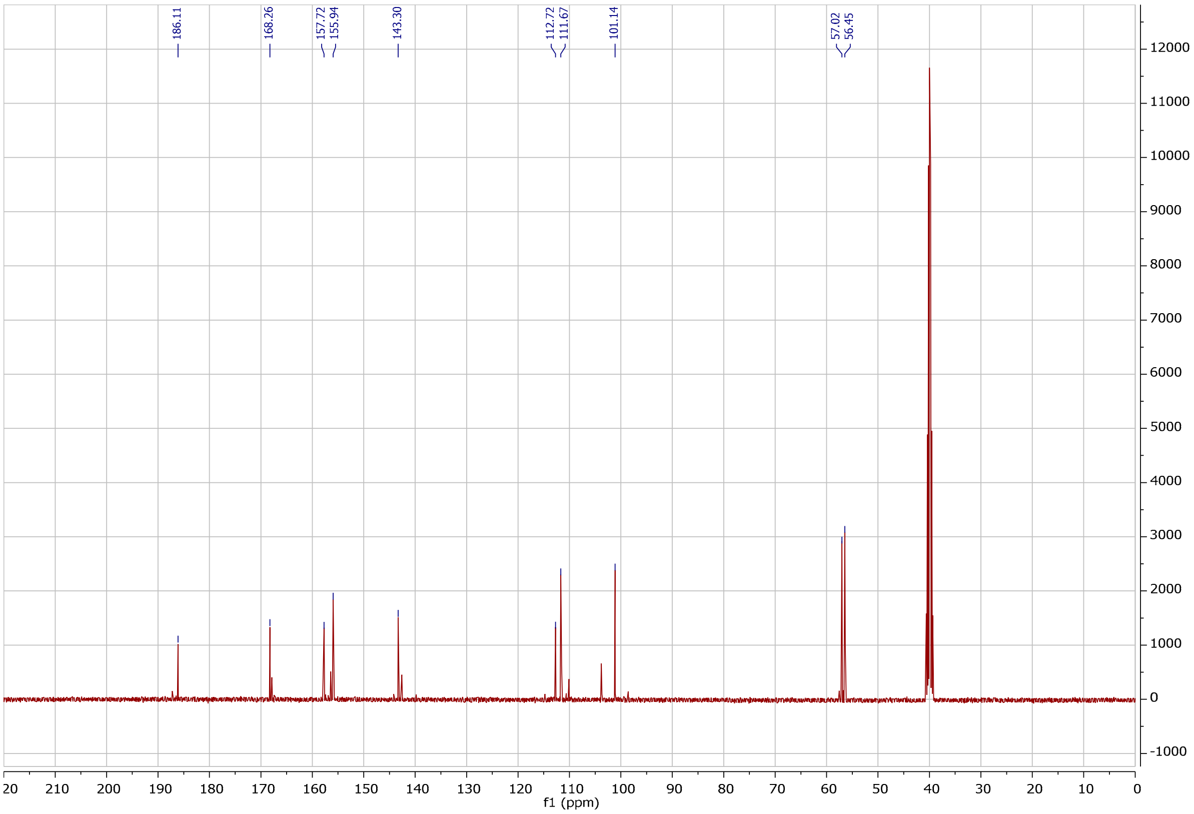
186.11
168.26
157.72
155.94
143.30
112.72
111.67
101.14
57.02
56.45
f1 (ppm)

6

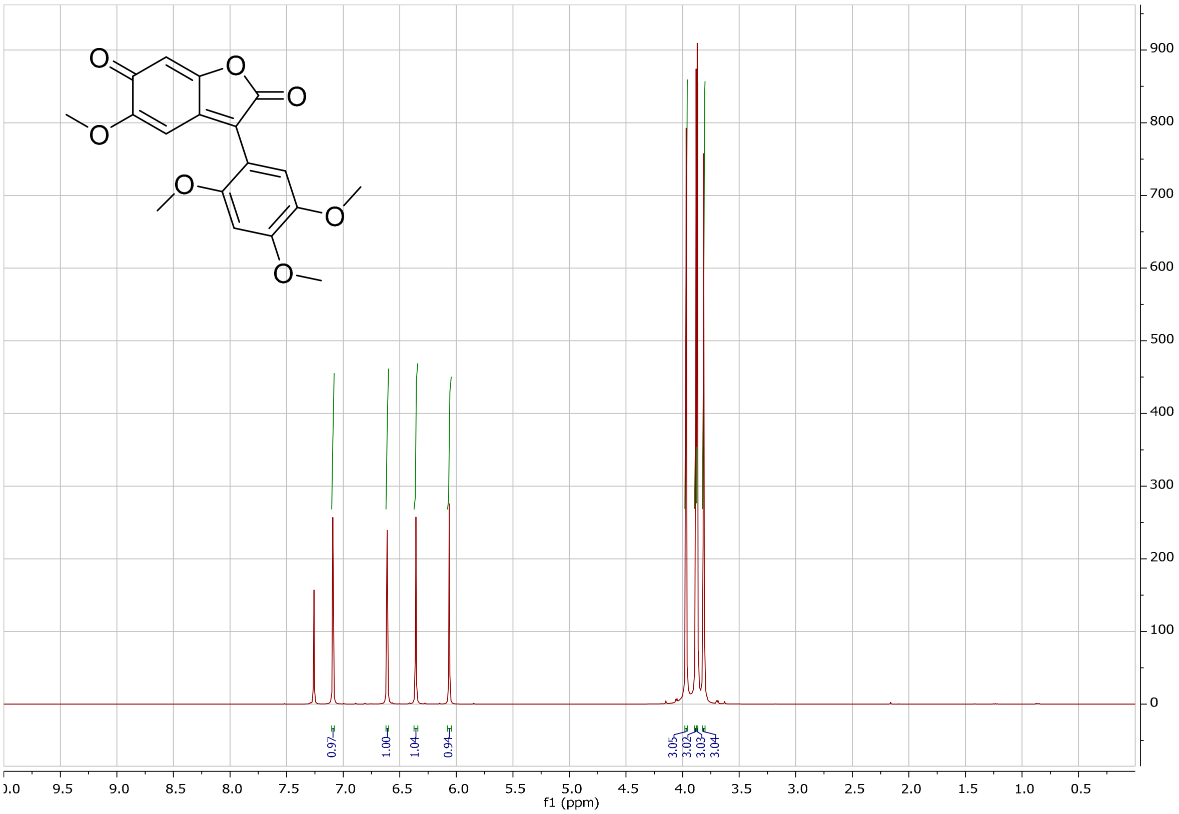
0.97
1.00
1.04
0.94
3.05
3.02
3.03
3.04
f1 (ppm)

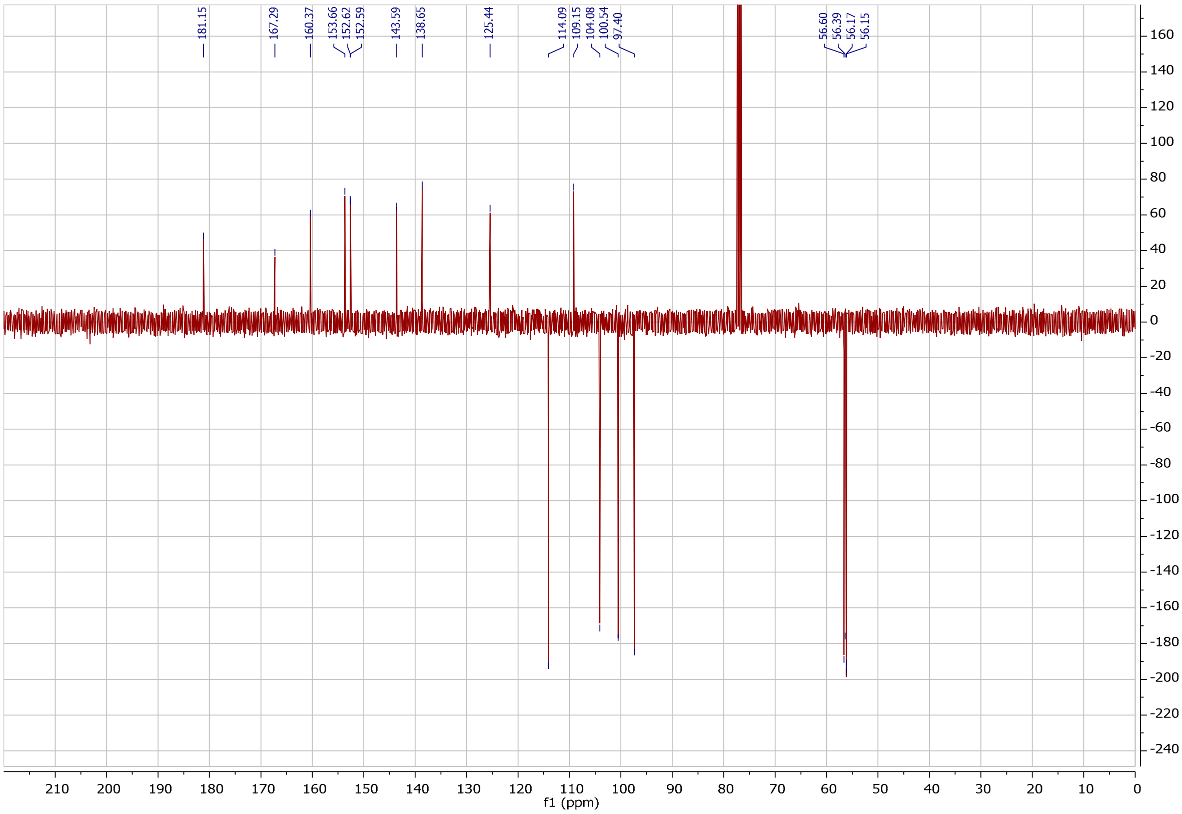
181.15
167.29
160.37
153.66
152.62
152.59
143.59
138.65
125.44
114.09
109.15
104.08
100.54
97.40
56.60
56.39
56.17
56.15
f1 (ppm)

9

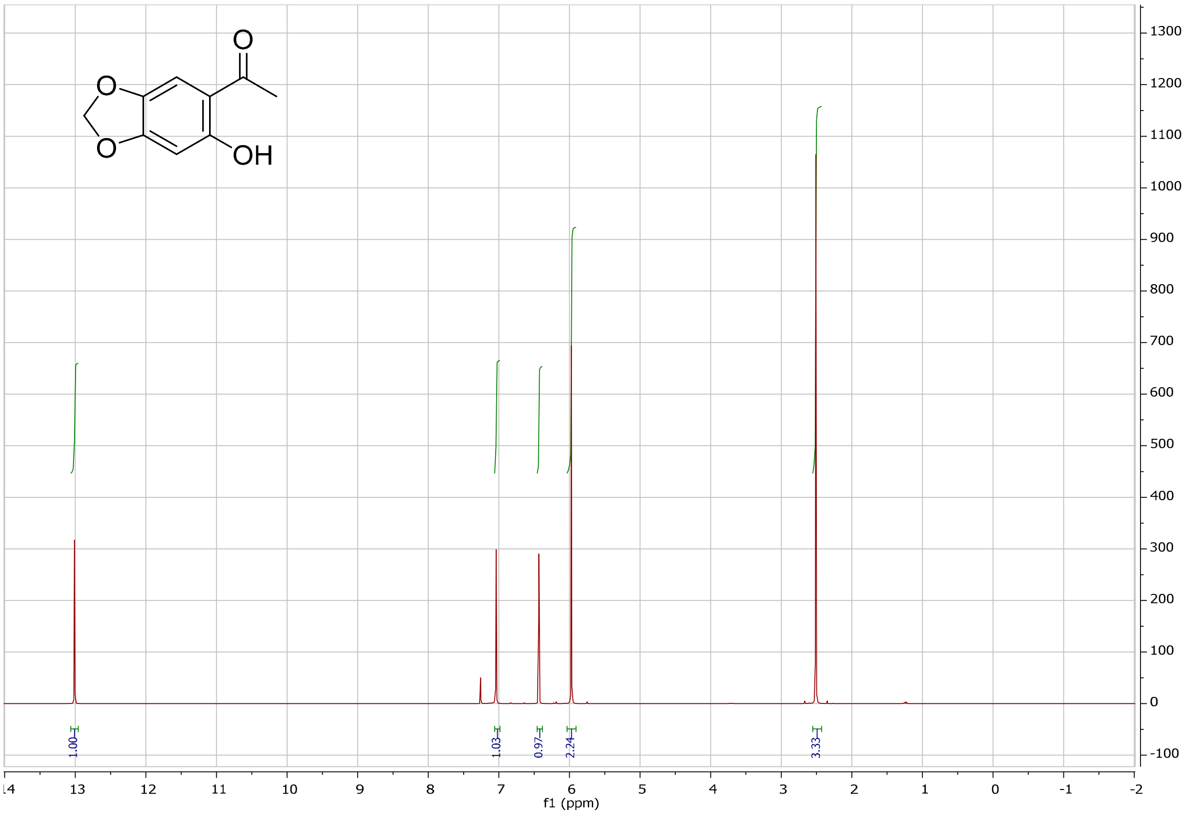
O
O
O
OH
1.00
1.03
0.97
2.24
3.33
f1 (ppm)

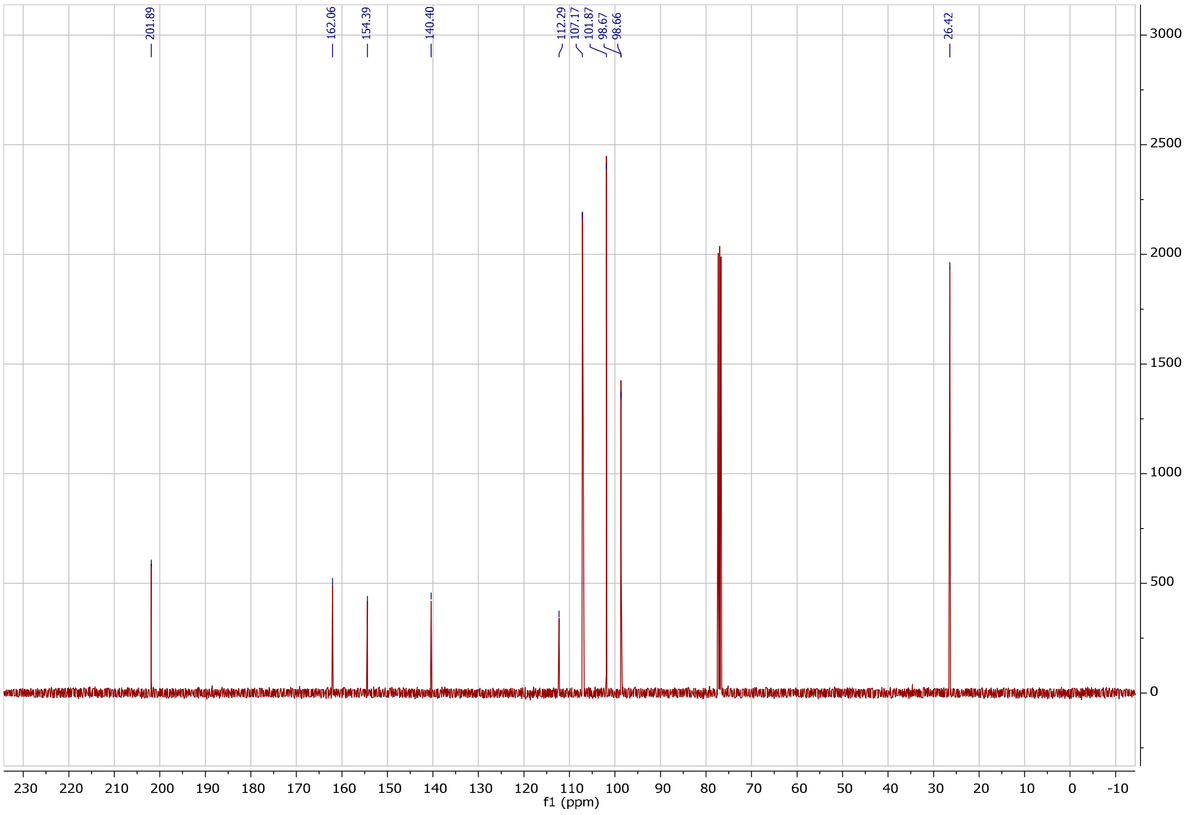
201.89
162.06
154.39
140.40
112.29
107.17
101.87
98.67
98.66
26.42
f1 (ppm)

10

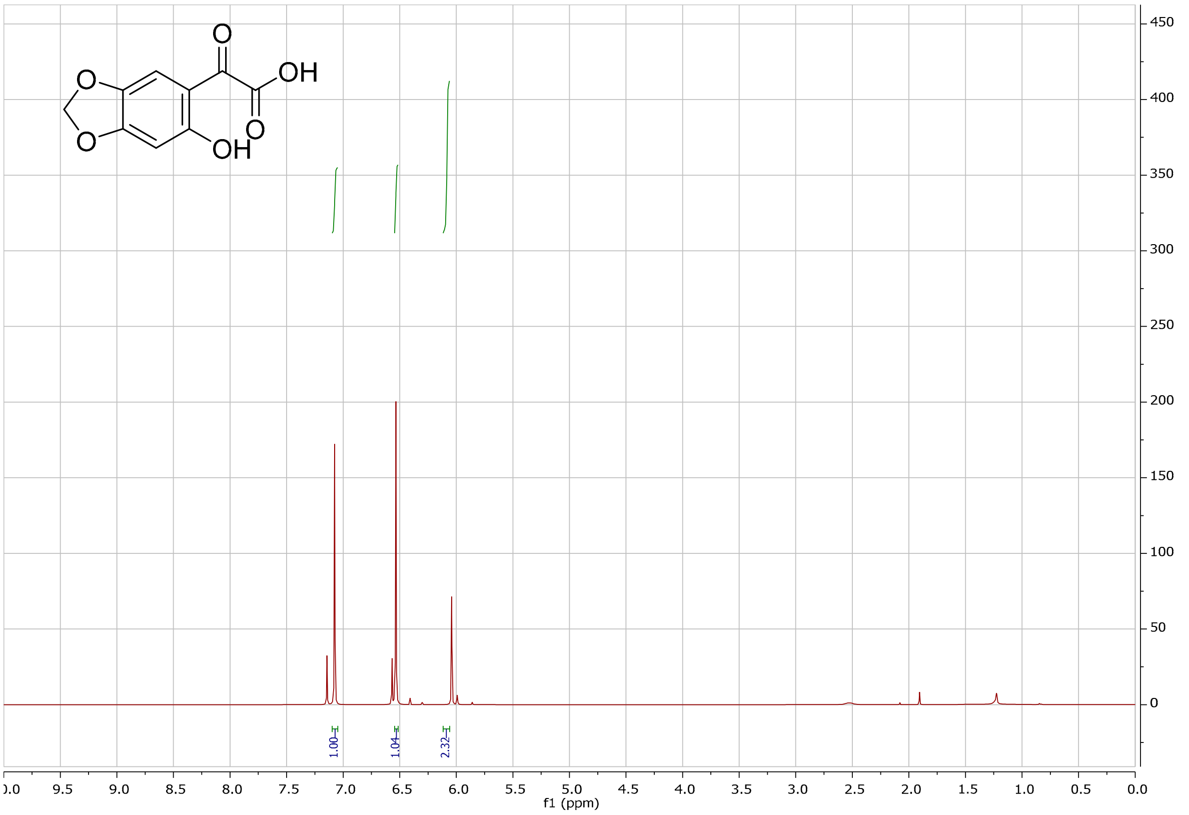
O
OH
O
O
O
OH
1.00
1.04
2.32
f1 (ppm)

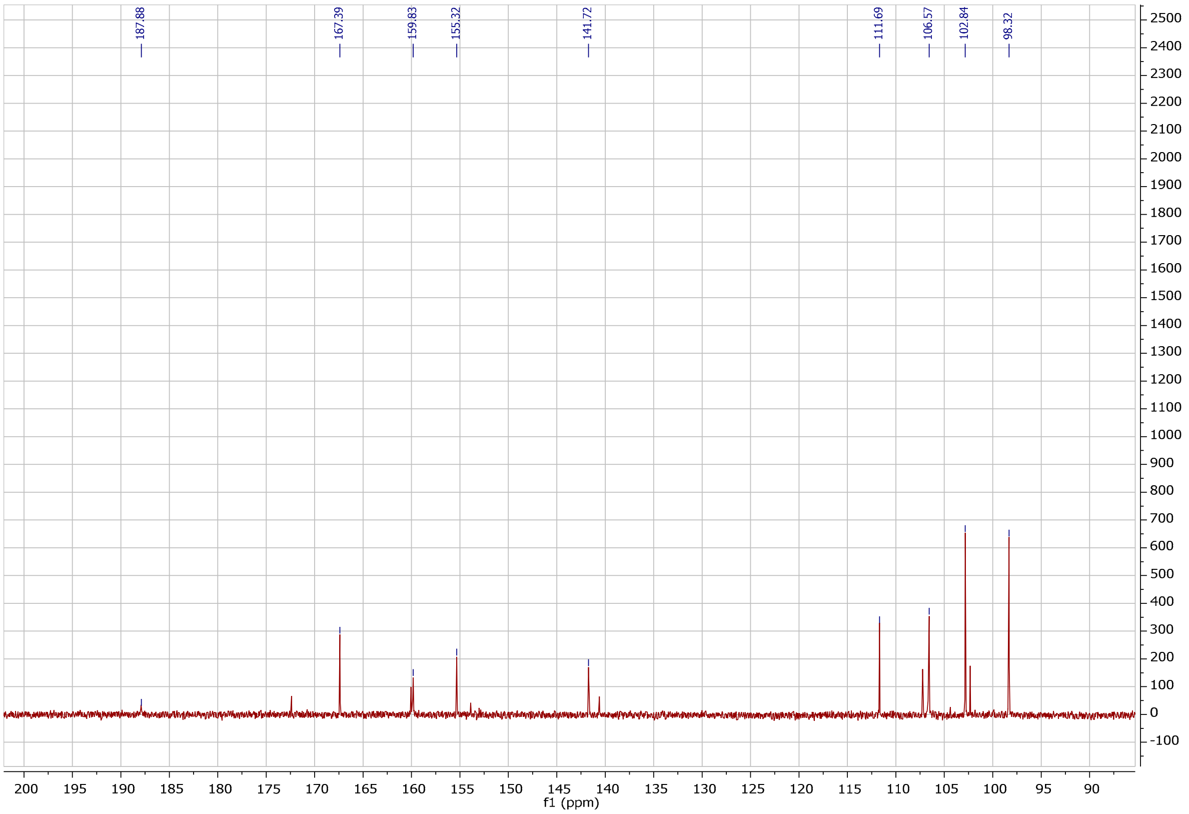
187.88
167.39
159.83
155.32
141.72
111.69
106.57
102.84
98.32
f1 (ppm)

11

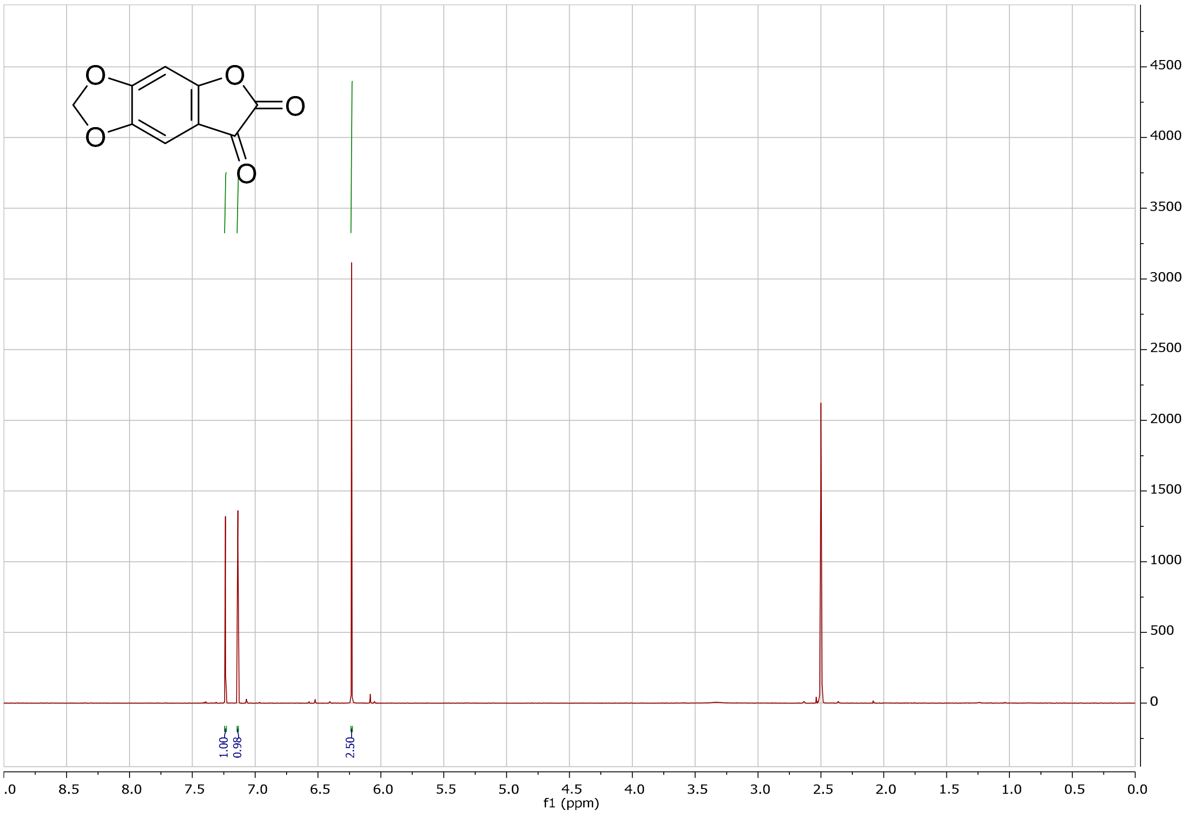
1.00
0.98
2.50
f1 (ppm)

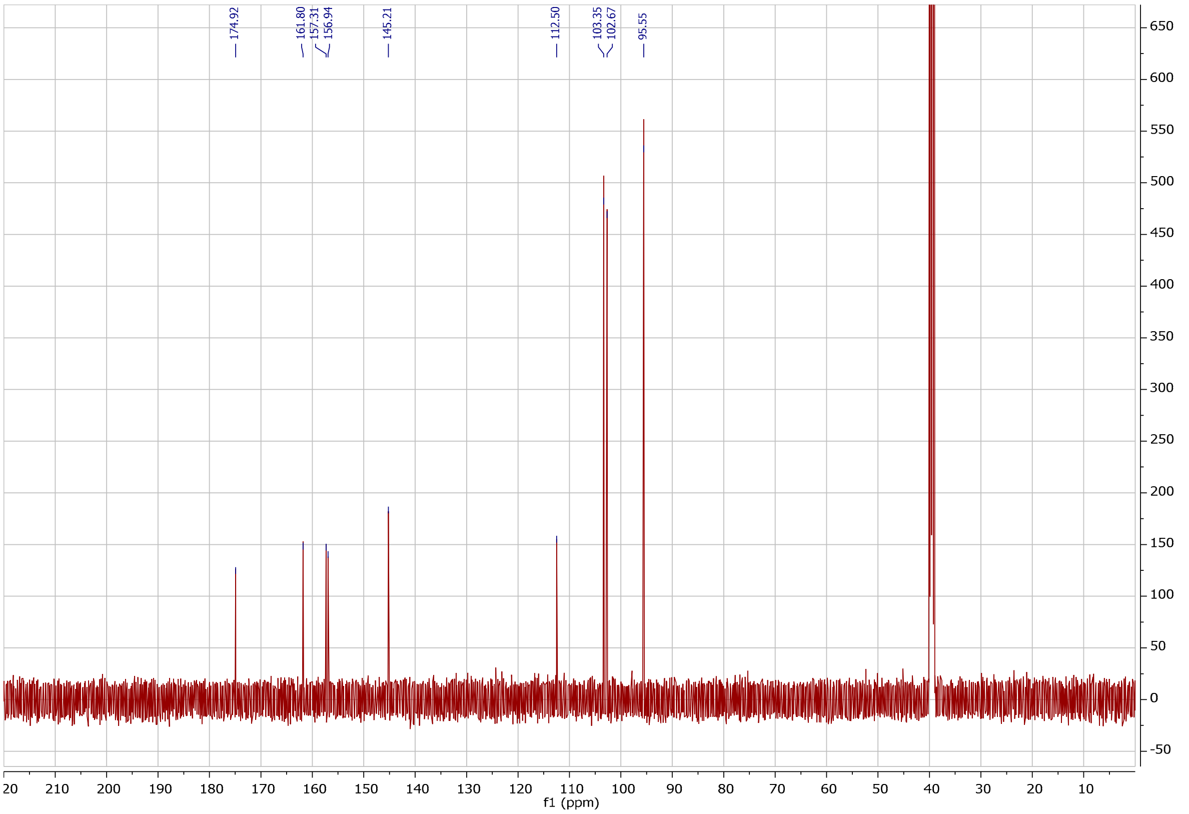
174.92
161.80
157.31
156.94
145.21
112.50
103.35
102.67
95.55
f1 (ppm)

12

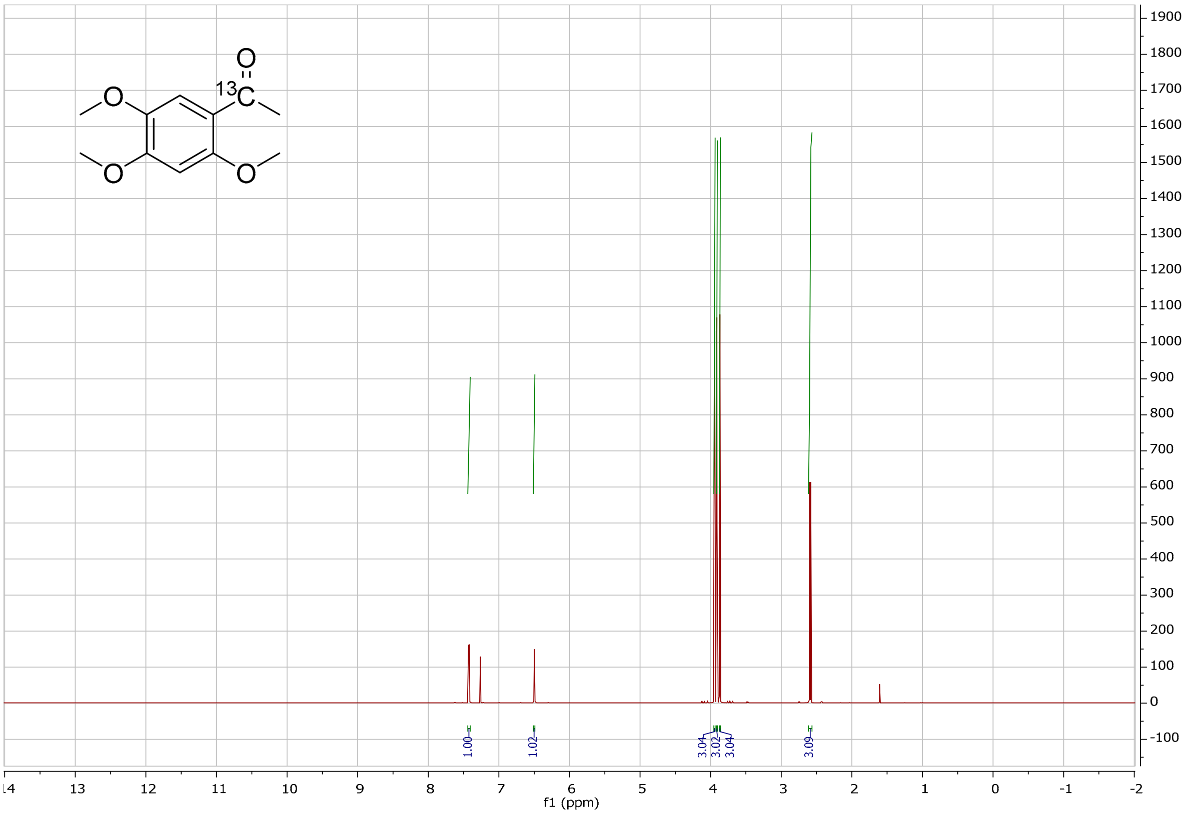
13C
1.00
1.02
3.04
3.02
3.04
3.09
f1 (ppm)

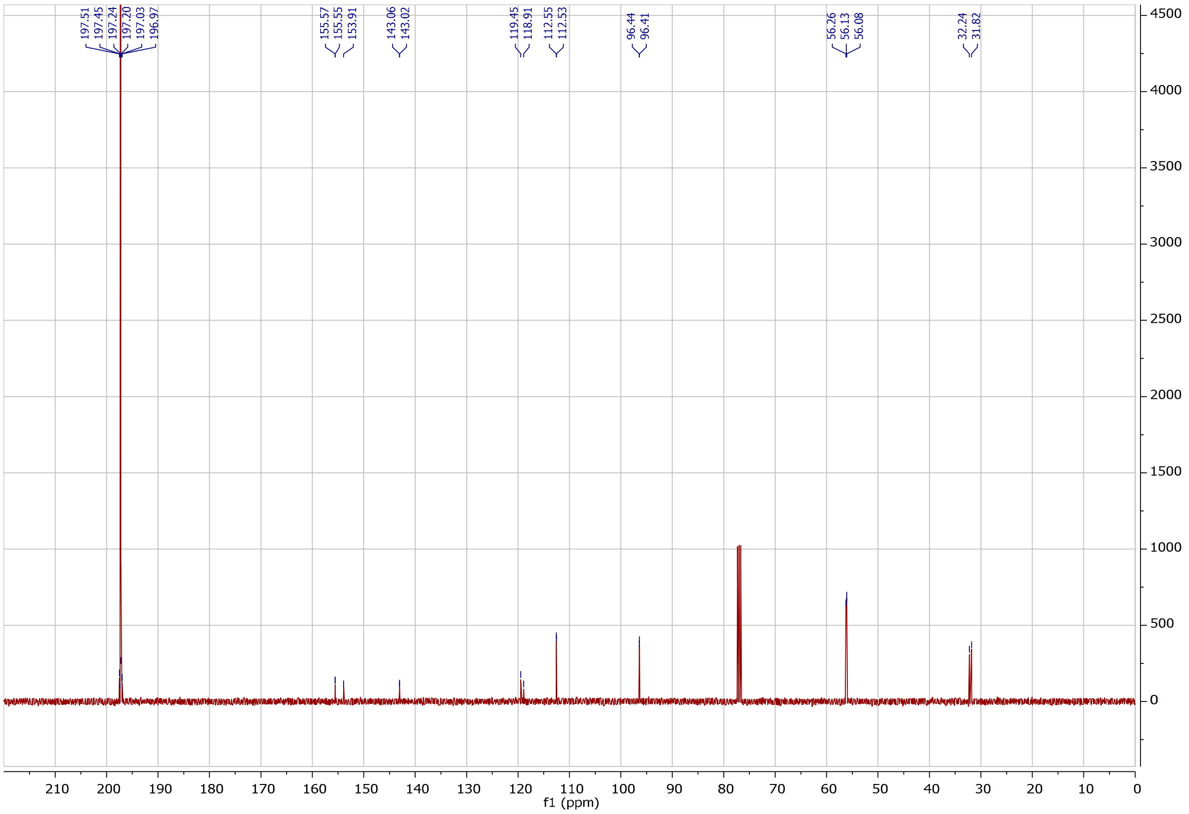
197.51
197.45
197.24
197.20
197.03
196.97
155.57
155.55
153.91
143.06
143.02
119.45
118.91
112.55
112.53
96.44
96.41
56.26
56.13
56.08
32.24
31.82
f1 (ppm)

13

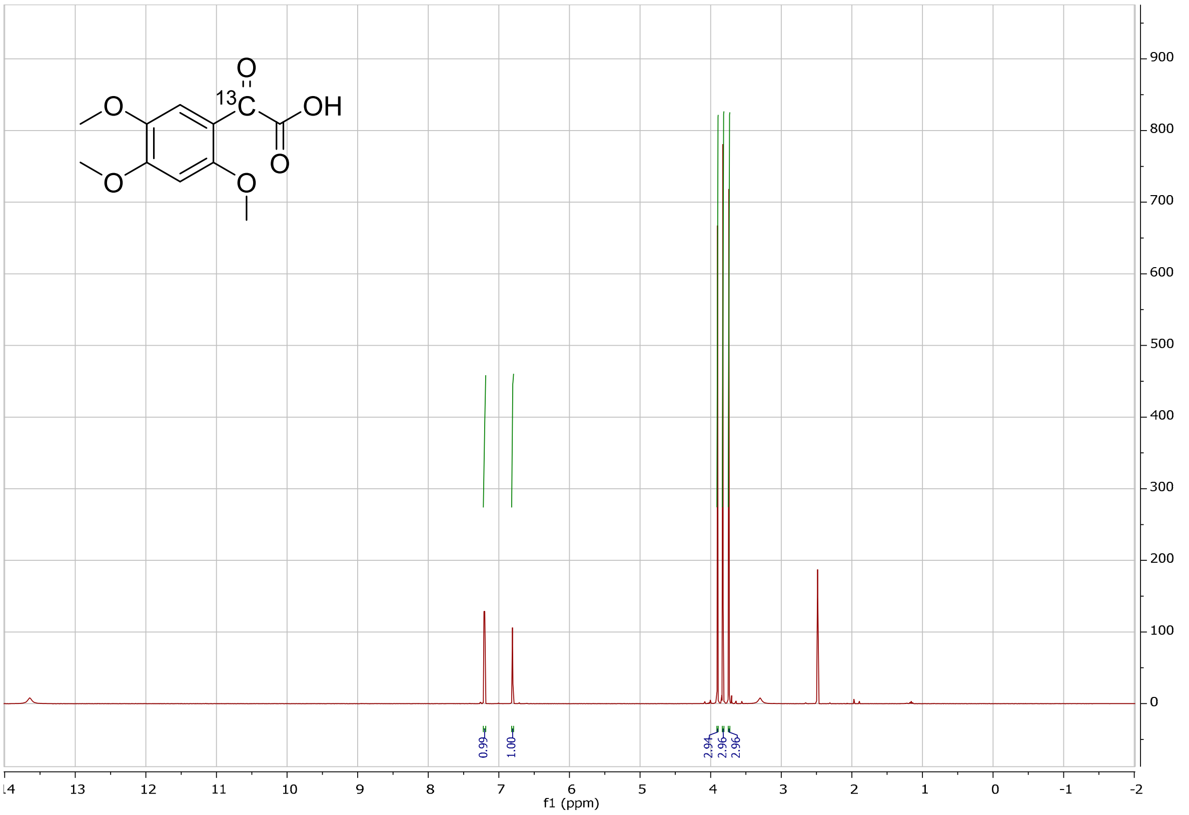

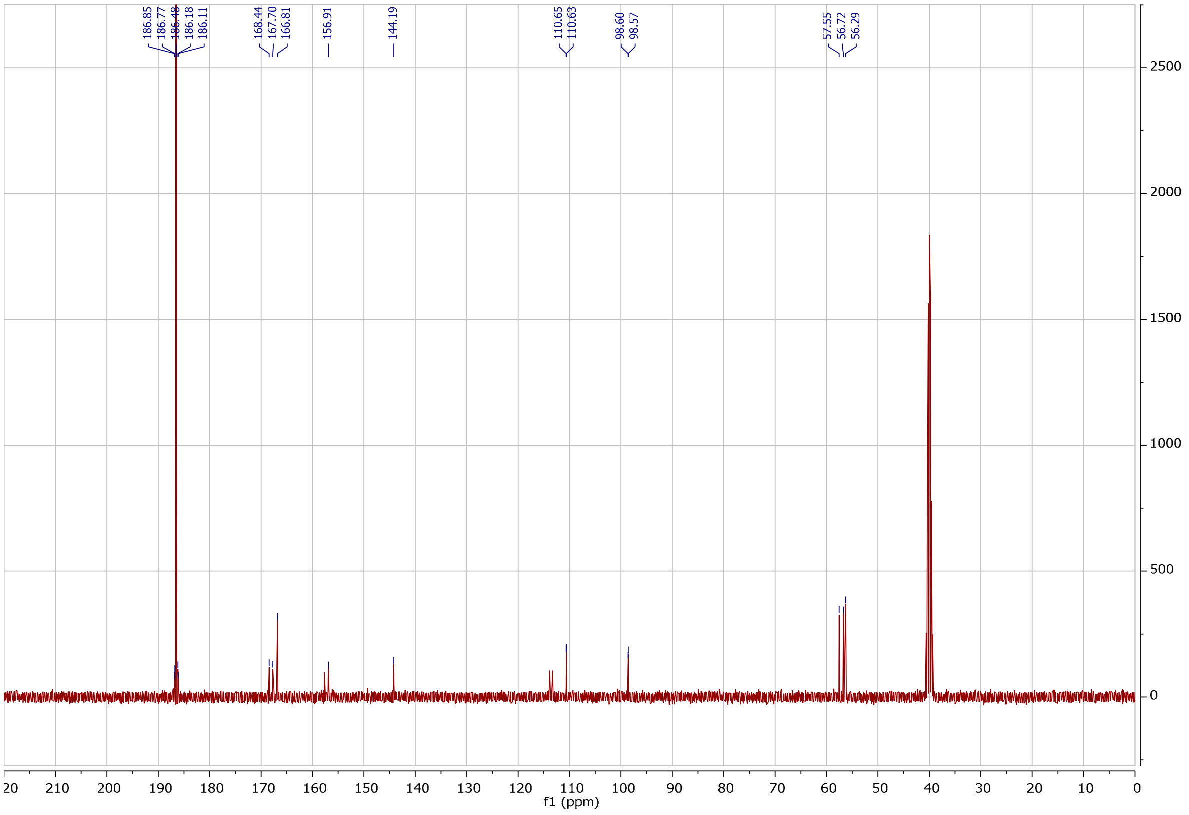

15

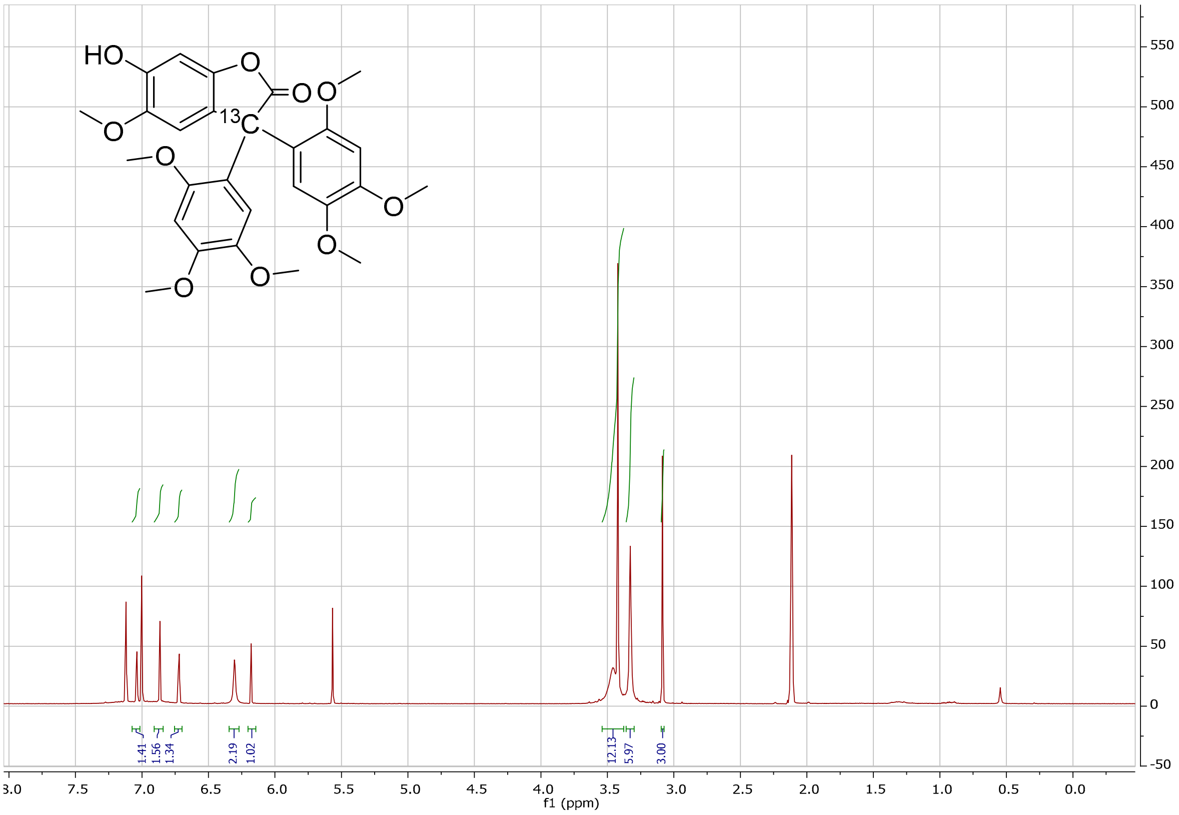
HO
O
O
O
O
13C
O
O
O
O
O
1.41
1.56
1.34
2.19
1.02
12.13
5.97
3.00
f1 (ppm)

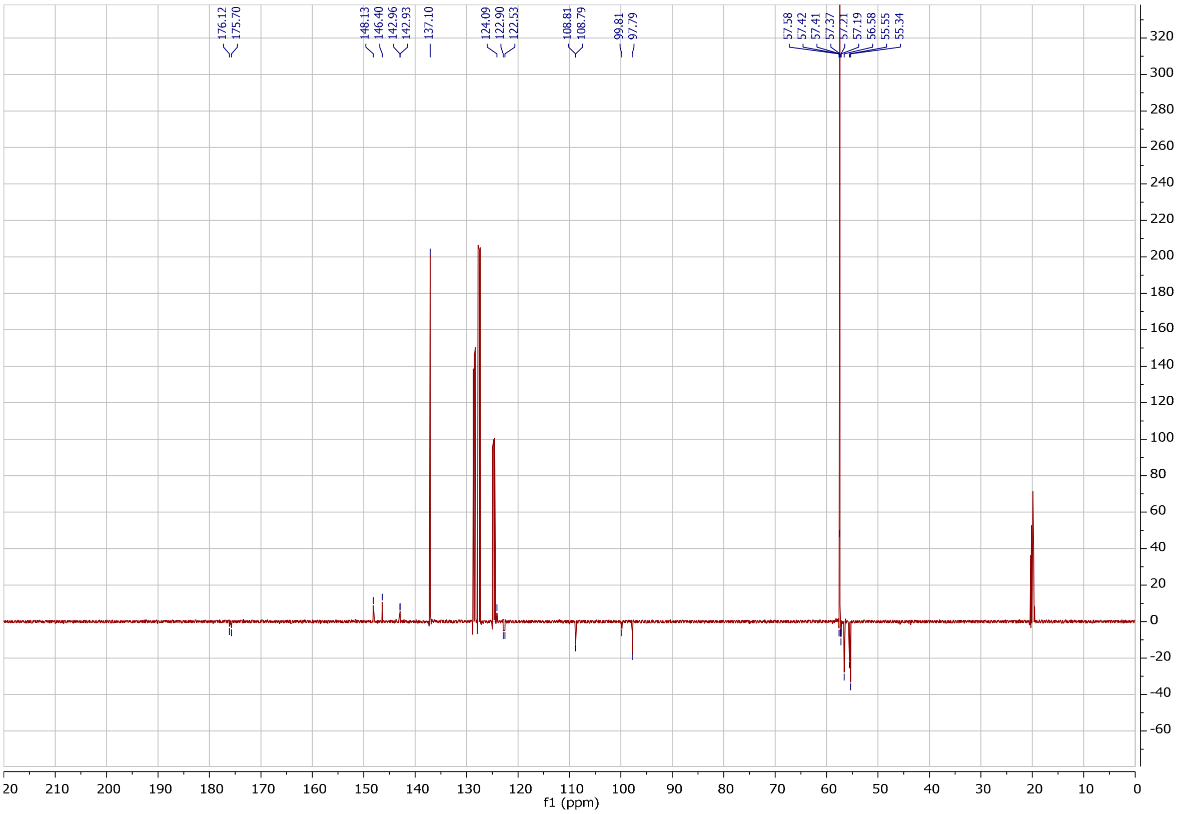
176.12
175.70
148.13
146.40
142.96
142.93
137.10
124.09
122.90
122.53
108.81
108.79
99.81
97.79
57.58
57.42
57.41
57.37
57.21
57.19
56.58
55.55
55.34
f1 (ppm)

17

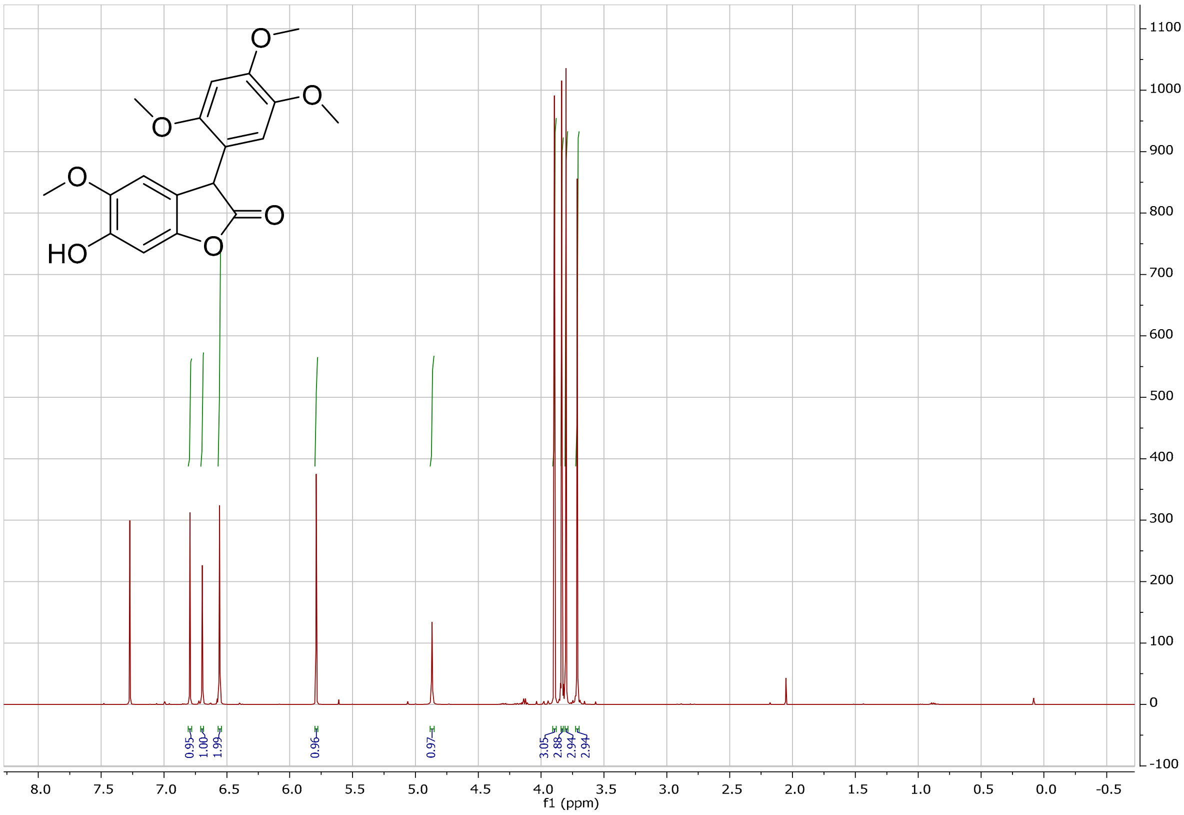
HO
O
0.95
1.00
1.99
0.96
0.97
3.05
2.88
2.94
2.94
f1 (ppm)

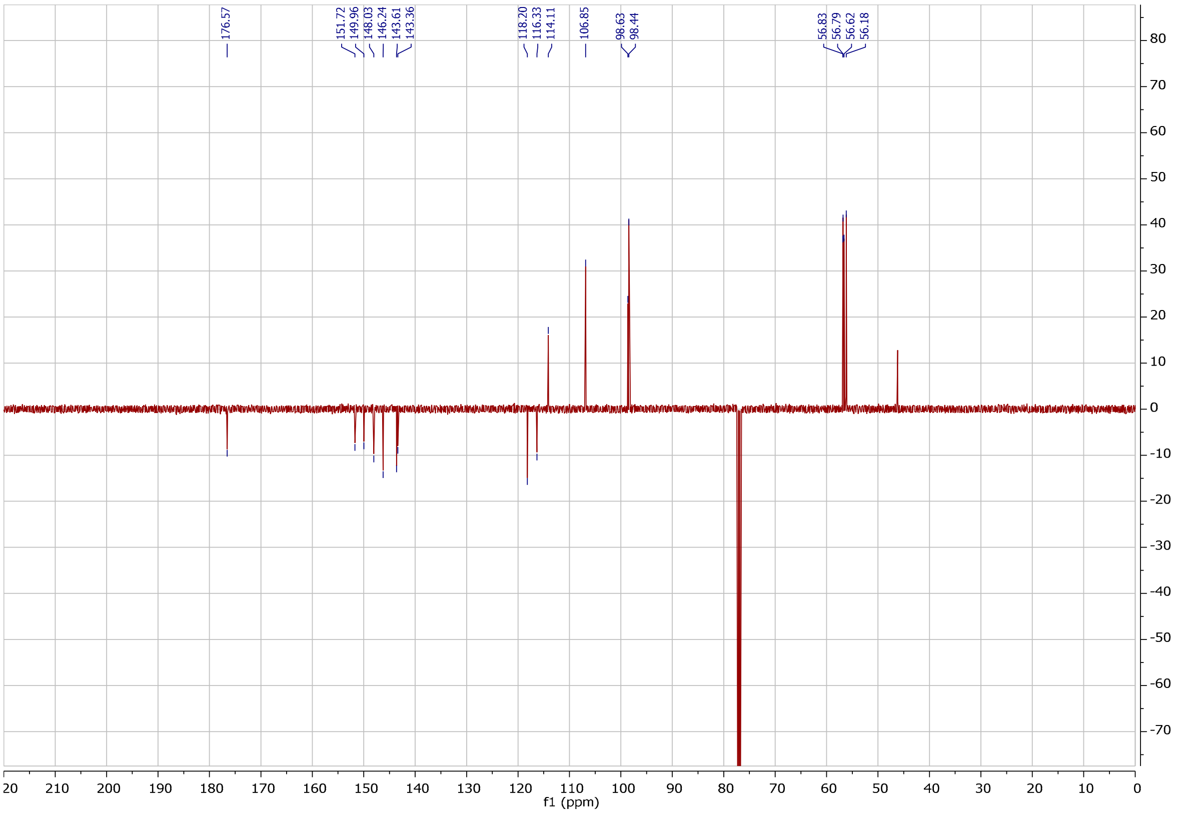
176.57
151.72
149.96
148.03
146.24
143.61
143.36
118.20
116.33
114.11
106.85
98.63
98.44
56.83
56.79
56.62
56.18
f1 (ppm)

18

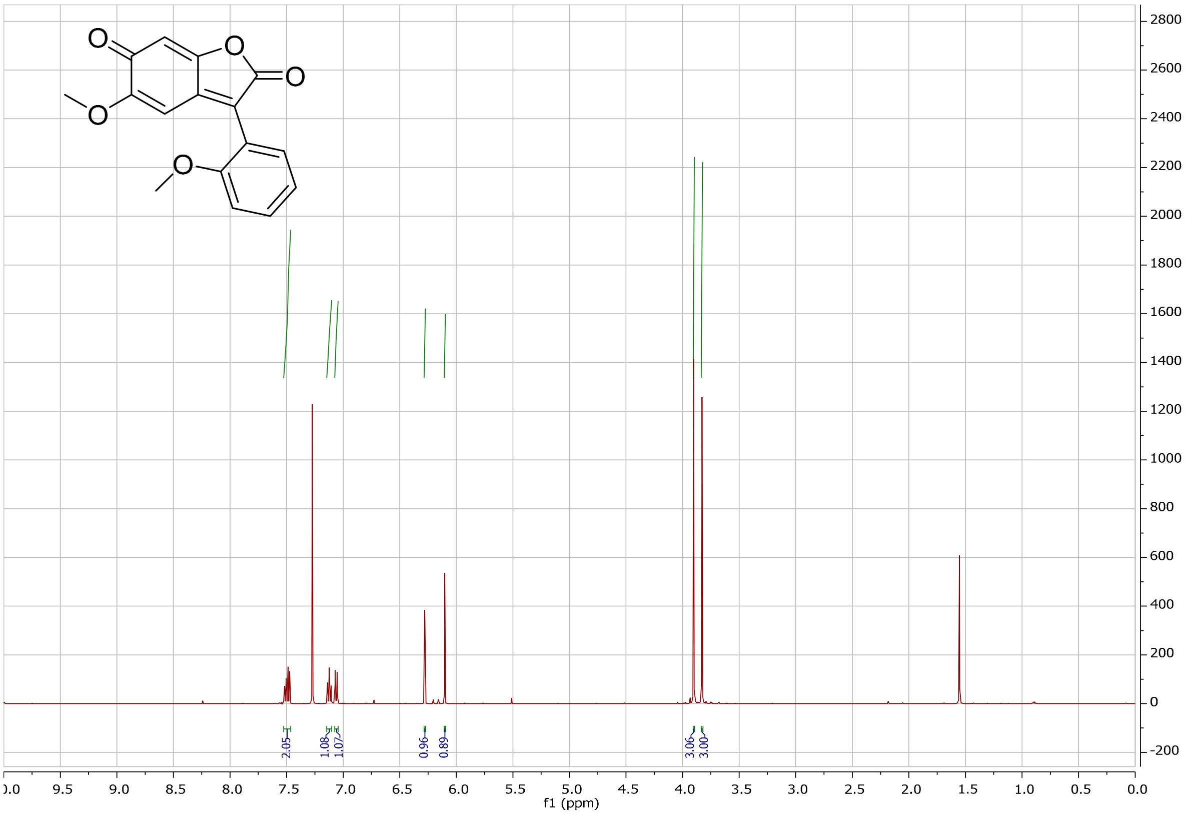
2.05
1.08
1.07
0.96
0.89
3.06
3.00
f1 (ppm)

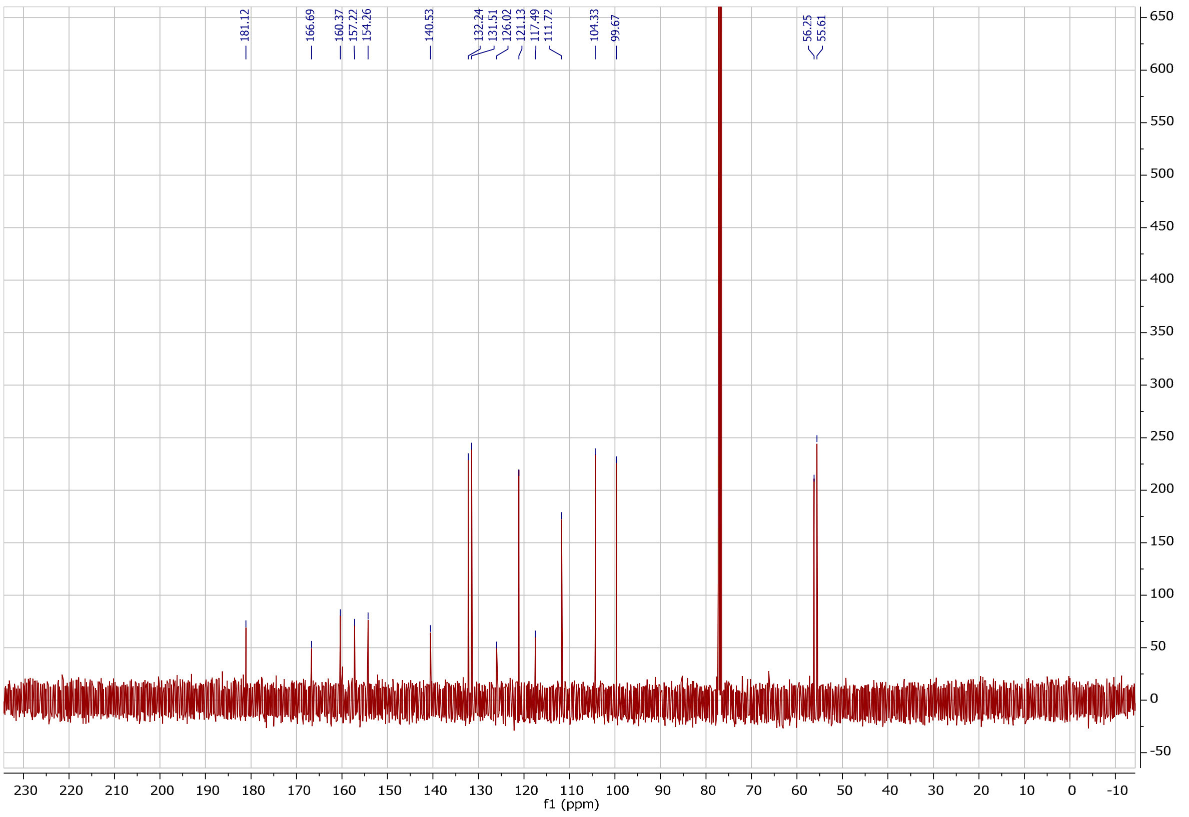
181.12
166.69
160.37
157.22
154.26
140.53
132.24
131.51
126.02
121.13
117.49
111.72
104.33
99.67
56.25
55.61
f1 (ppm)

19

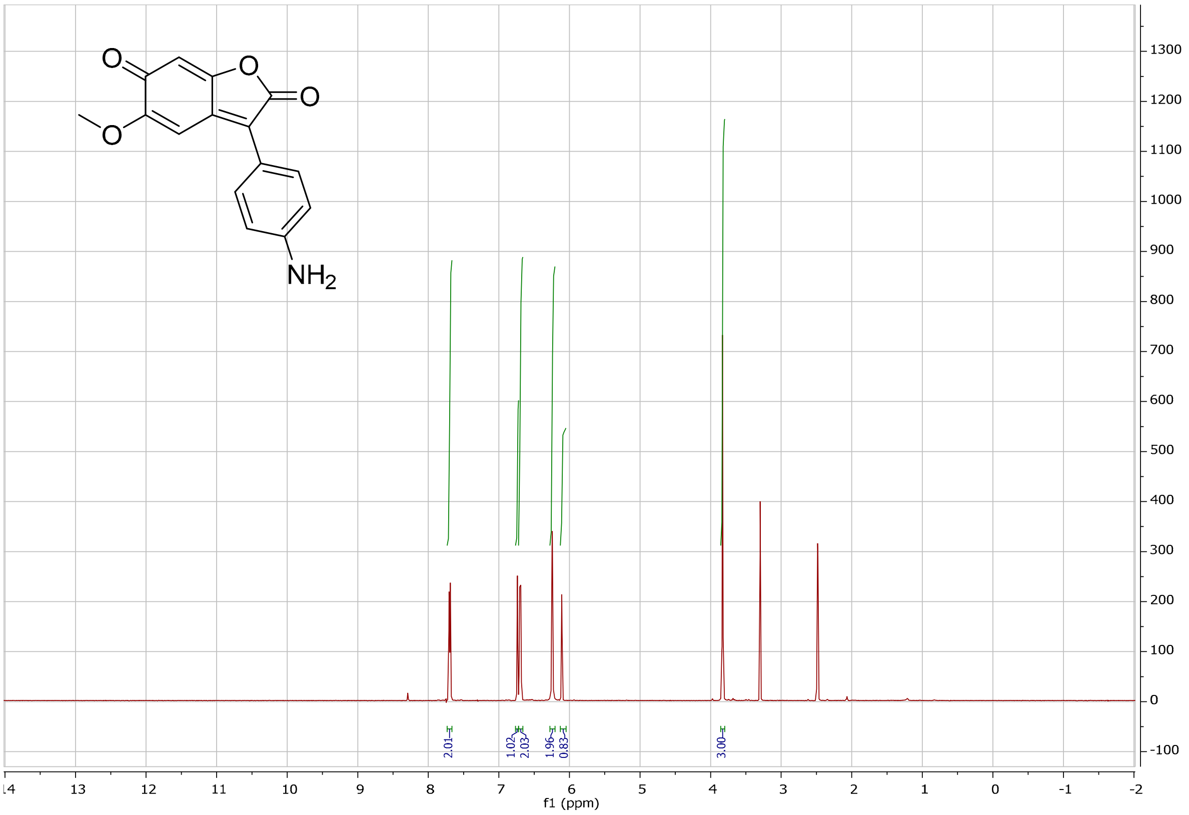
O
O
O
O
NH2
2.01
1.02
2.03
1.96
0.83
3.00
f1 (ppm)

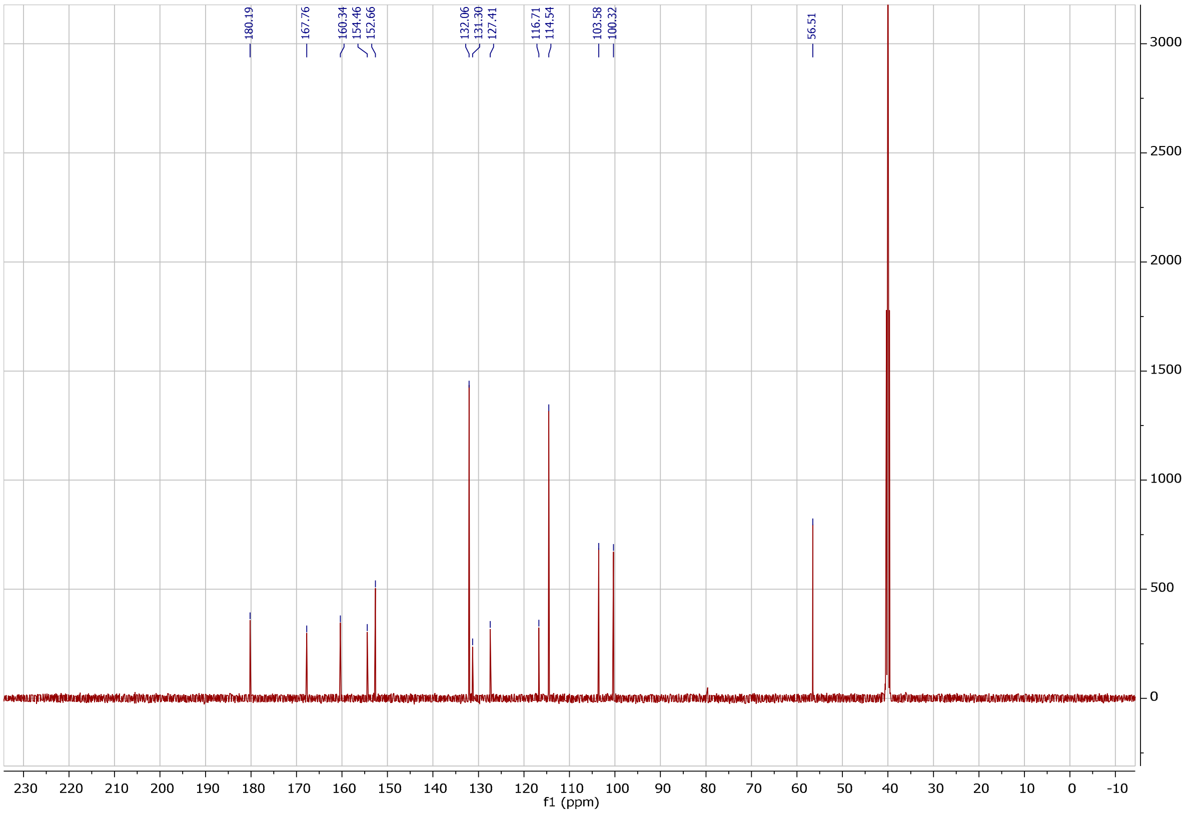
180.19
167.76
160.34
154.46
152.66
132.06
131.30
127.41
116.71
114.54
103.58
100.32
56.51
f1 (ppm)

20

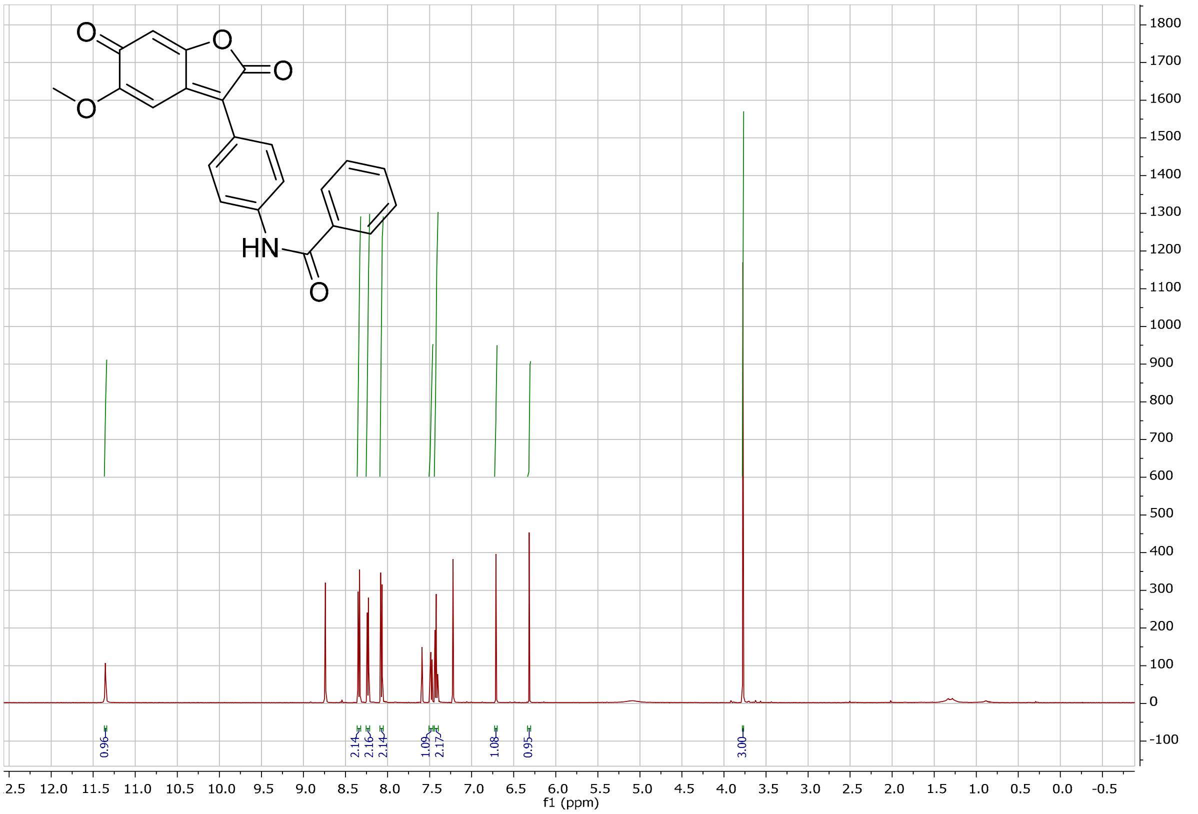
O
O
O
O
HN
O
0.96
2.14
2.16
2.14
1.09
2.17
1.08
0.95
3.00
f1 (ppm)

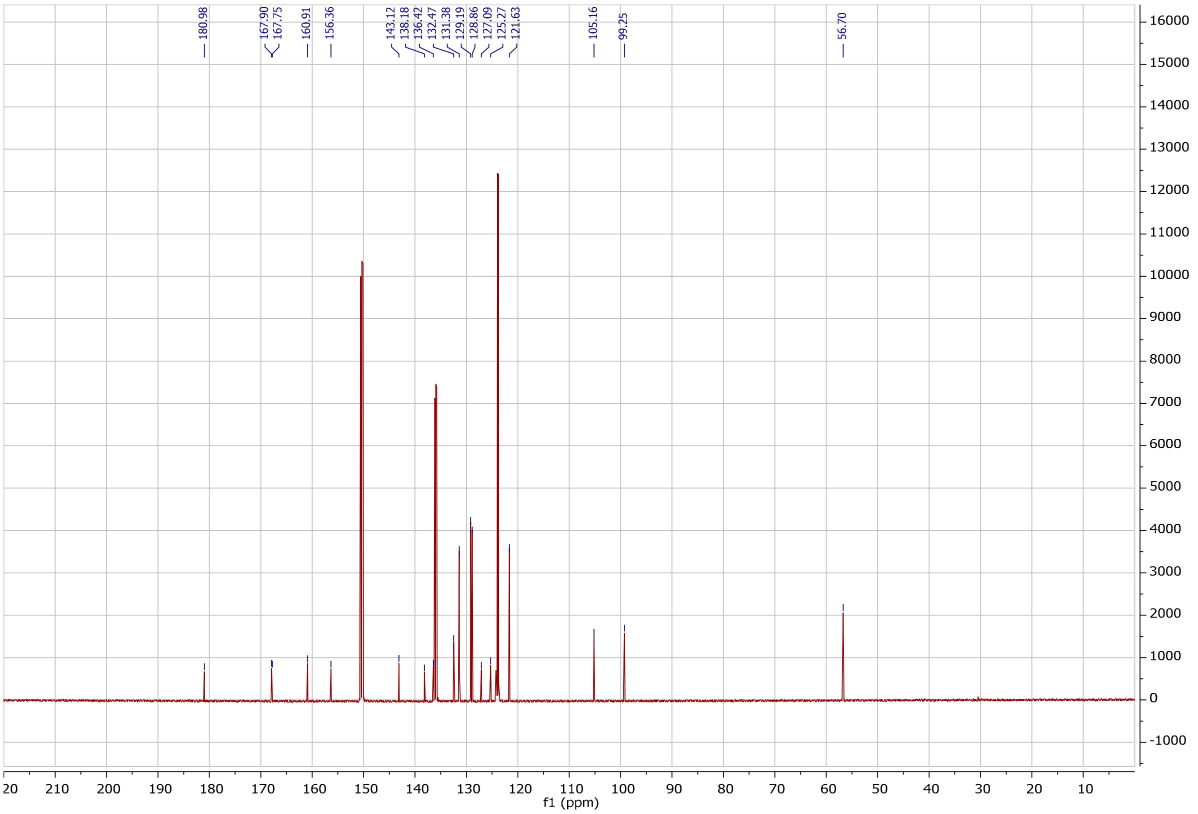
180.98
167.90
167.75
160.91
156.36
143.12
138.18
136.42
132.47
131.38
129.19
128.86
127.09
125.27
121.63
105.16
99.25
56.70
f1 (ppm)

21

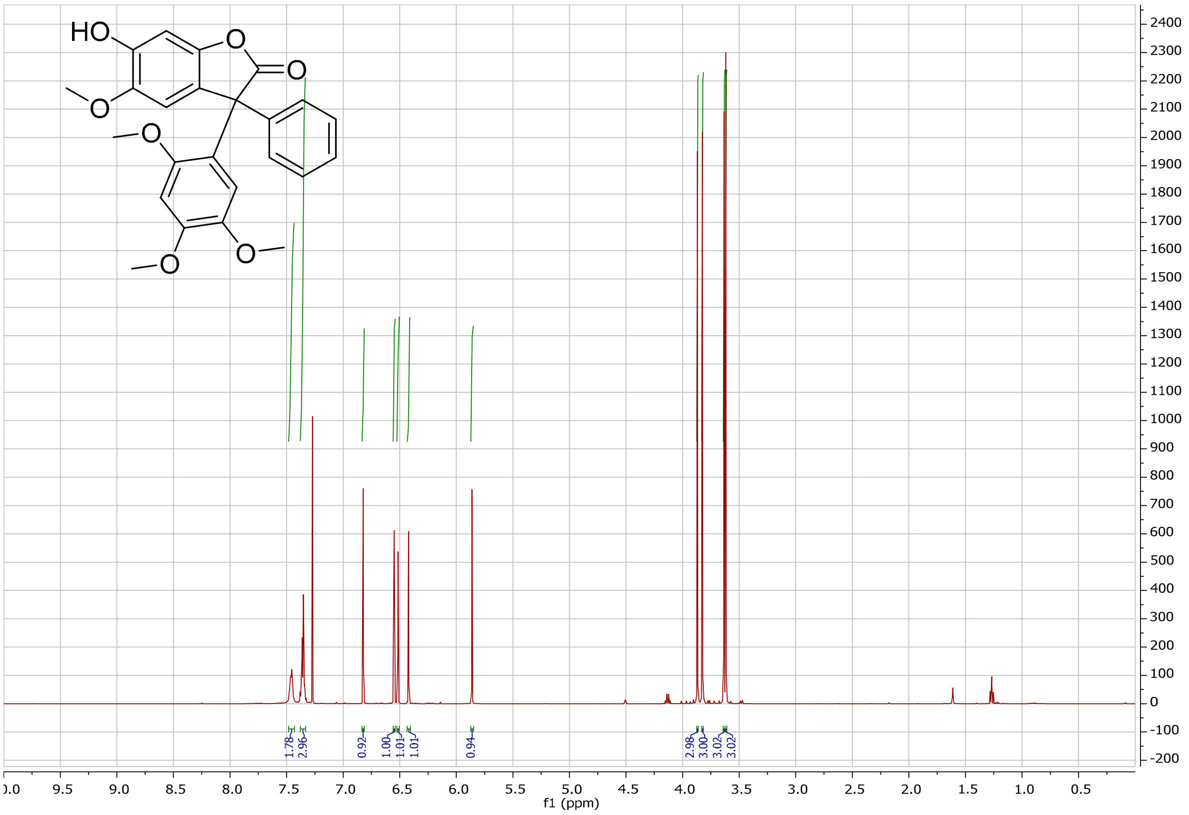
HO
O
O
O
O
O
O
1.78
2.96
0.92
1.00
1.01
1.01
0.94
2.98
3.00
3.02
3.02
f1 (ppm)

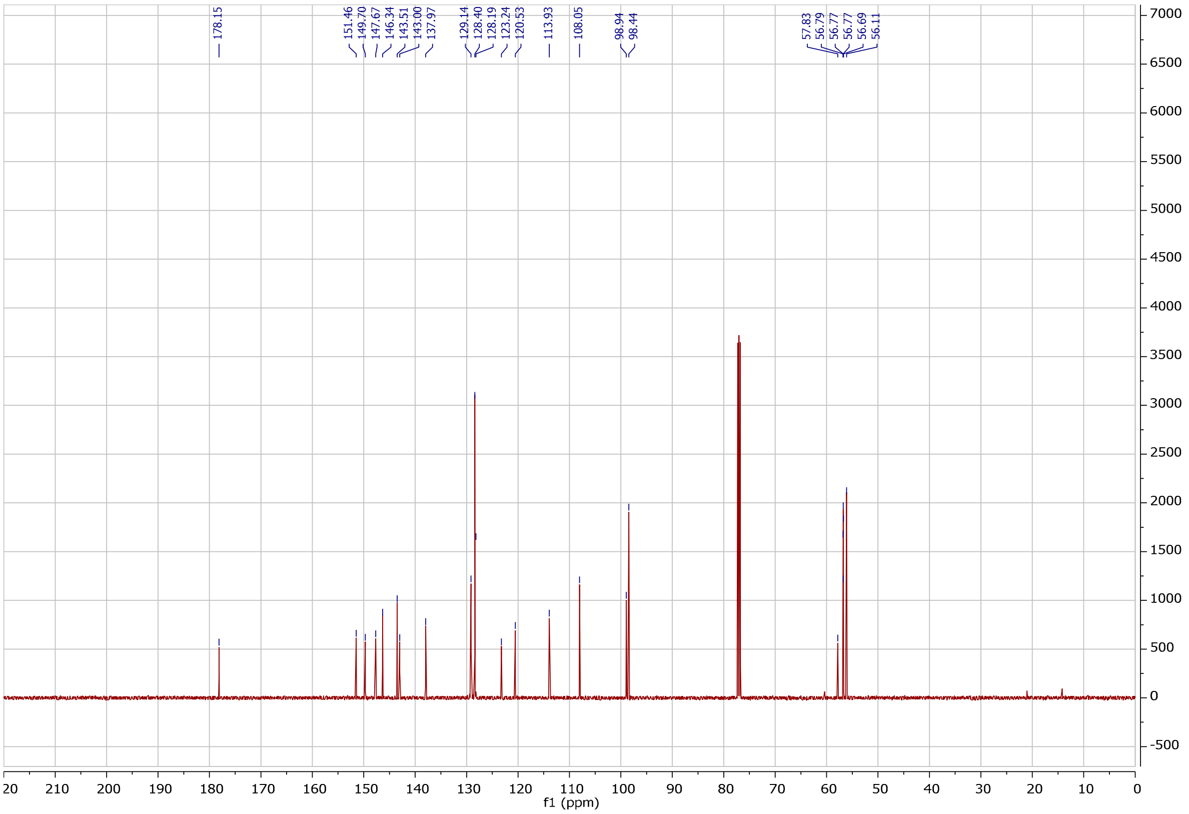
178.15
151.46
149.70
147.67
146.34
143.51
143.00
137.97
129.14
128.40
128.19
123.24
120.53
113.93
108.05
98.94
98.44
57.83
56.79
56.77
56.69
56.11
f1 (ppm)
